# Supplementary material for: Dorsal prefrontal cortex drives perseverative behavior in mice
Source: Nat Commun. 2026 Apr 25;17:5715. doi: 10.1038/s41467-026-71664-w (PMC13324276; doi:10.1038/s41467-026-71664-w)
Supplement: Supplementary file 1 — Supplementary Information [file 41467_2026_71664_MOESM1_ESM.pdf]

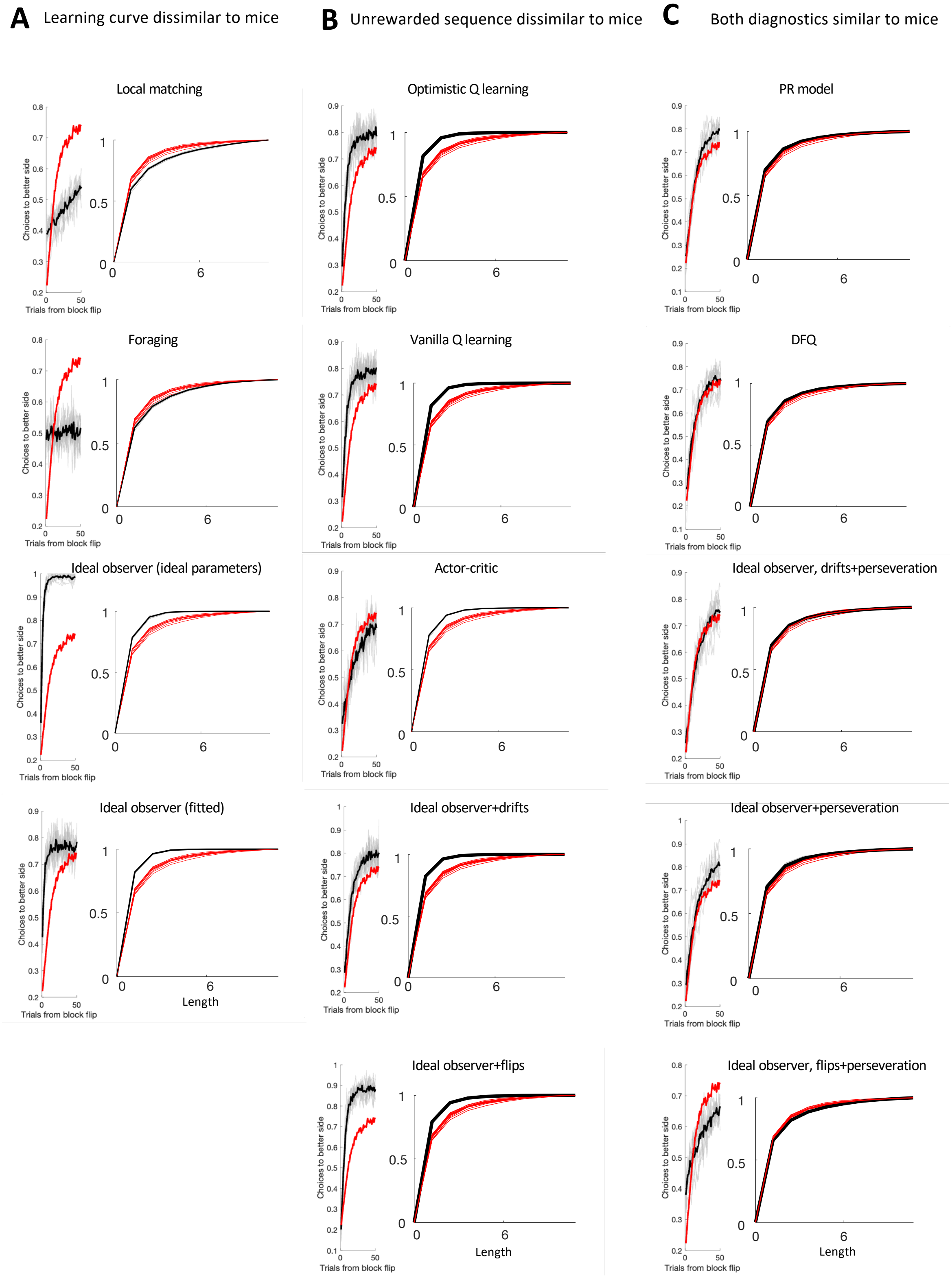

Supplementary figure 1. Diagnostics for all models. Two behavioural diagnostic plots are shown for each model: the learning curve (fraction of choices to the correct side as a function of time since a block switch, as in Fig. 1D); cumulative proportion of the lengths of unrewarded sequences, as in Fig. 1E. Grey lines, data for individual fits; black line, average. A, Models in which the first diagnostic is dissimilar to mice. B, Models in which the first diagnostic is dissimilar to mice. C, Models in which both diagnostics are similar to mouse diagnostics. Note, all models in C are perseverative. Analyses were performed in MATLAB R2023a (The MathWorks, Natick, MA, USA).

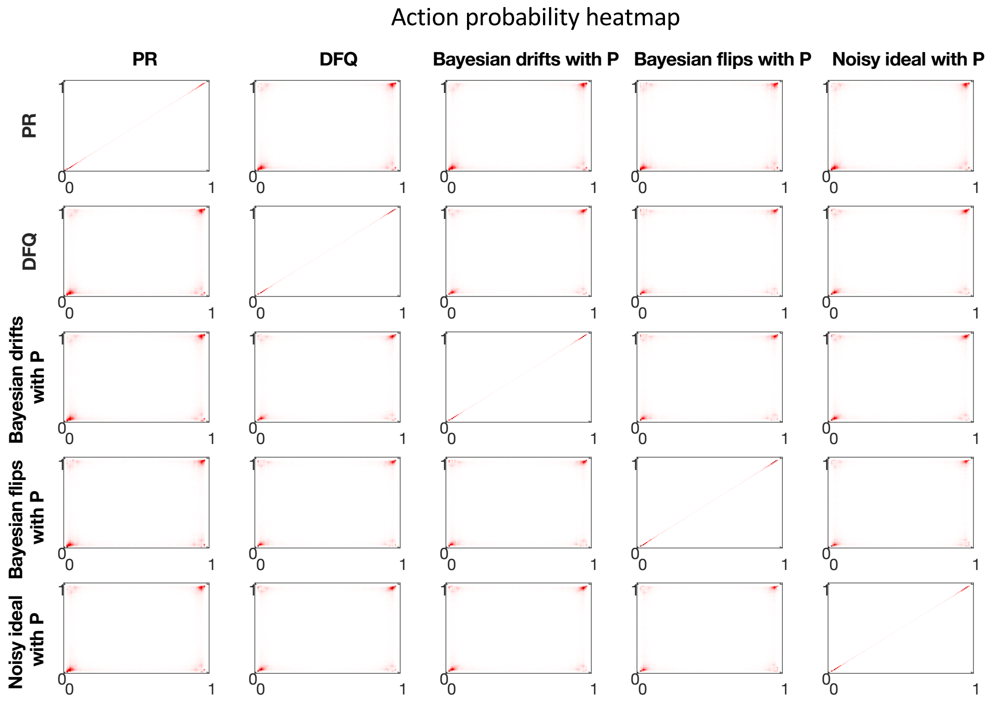

Supplementary figure 2. Similarity of choice predictions for the 5 winning models. Each panel shows a pseudocolor histogram summarizing the frequency that every pair of choice probabilities was predicted by two models, across all trials, sessions, and mice. For example, the intensity in the top left corner of the top right plot shows the frequency of trials on which the PR model predicted p(Right)=0 and the ideal observer model predicted p(Right)=1. Analyses were performed in MATLAB R2023a (The MathWorks, Natick, MA, USA).

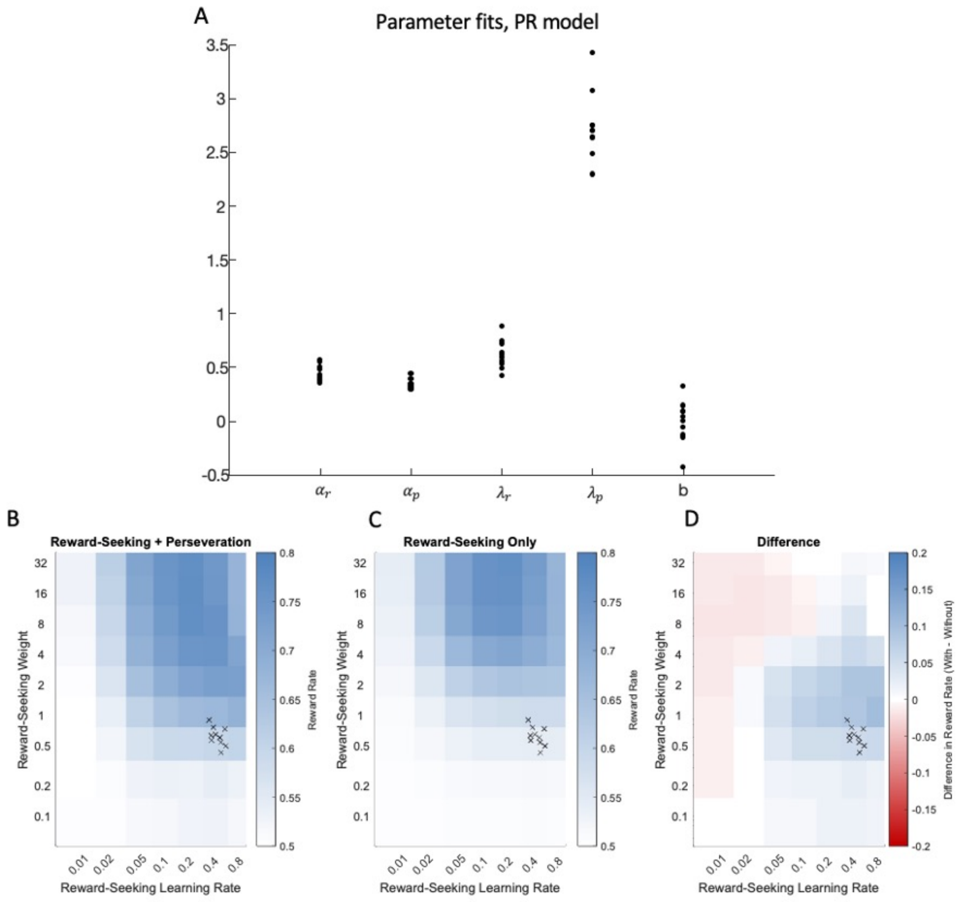

Supplementary figure 3 | Fit parameters: PR model. A, The parameter fits for the PR model for each mouse. B, reward rate obtained in simulations of the PR model, varying the reward seeking weight λ_r and reward learning rate α_r parameters, while maintaining the mean values of the perseveration variables α_p and λ_p fit to mouse behavior. Crosses indicate the values of α_r and λ_r fit to mouse data. The highest reward rates are obtained at much larger values of λ_r and slightly lower values of α_r than those used by the mice. C, same but for simulations with no perseveration ($\alpha_p = 0$), indicating the reward-rate that can be obtained by a reward-seeking agent on its own. D, difference between the left and middle panels. Including a perseverative agent with mouse-like parameters helps, rather than hinders, reward rate over much of the range. Analyses were performed in MATLAB R2023a (The MathWorks, Natick, MA, USA).

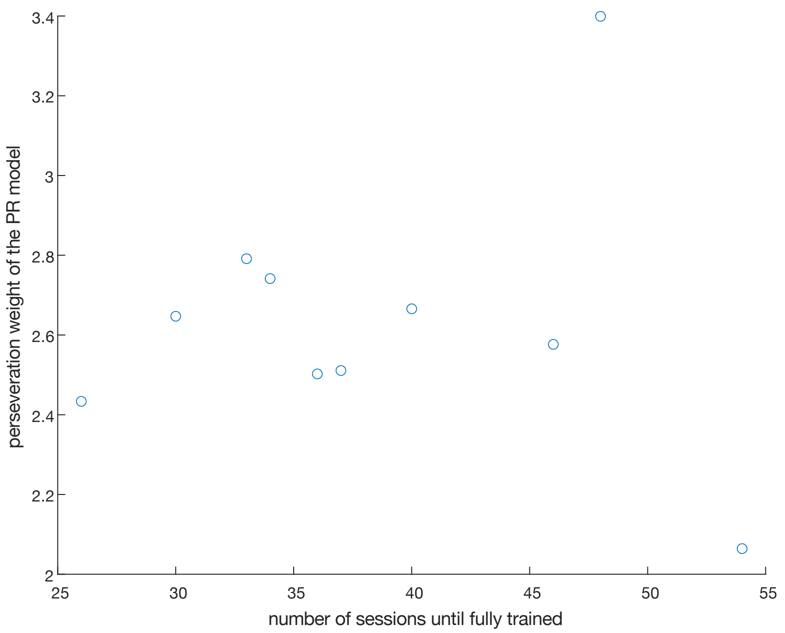

Supplementary figure 4. Number of sessions until animal was fully trained on the task against the perseveration weight in the PR model based on this animal's performance. The decision to call an animal fully trained was made by the experimenter subjectively and was based on the number of trials in each session, as well as the reward received. Analyses were performed in MATLAB R2023a (The MathWorks, Natick, MA, USA).

A

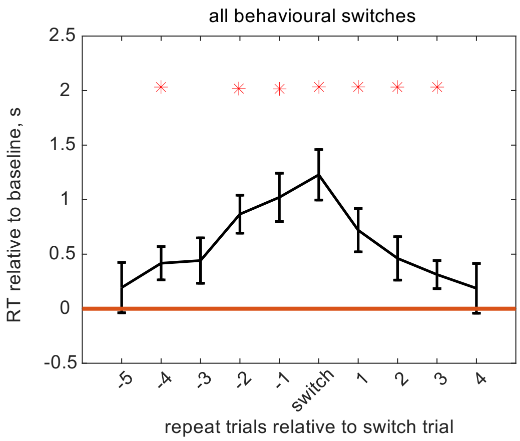

B

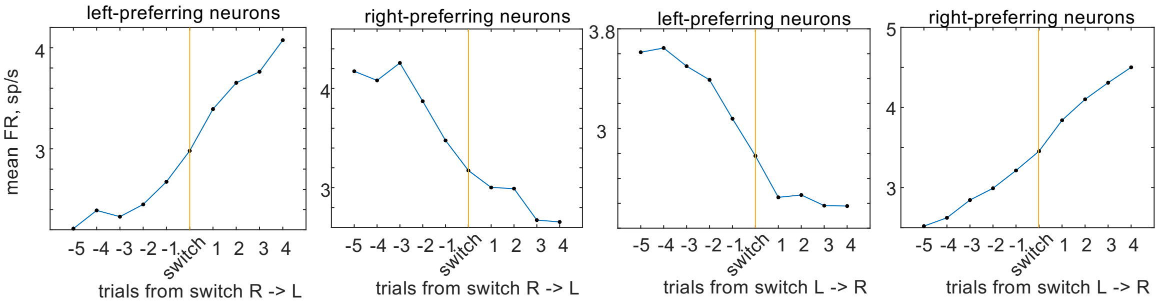

Supplementary figure 5 | Changes in response time and neural activity in MOs relative to behavior switches. A, median response time as a function of the number of trials relative to the choice switch, minus overall median response time. An x-axis value of n indicates median reaction times for all trials exactly n trials following a switch, without a second intervening switch. Thus the trial following the sequence LRRR would go into bin 3 only, while the last trial of sequence LRLR would go into bin 1 only. Red stars indicate a significant difference from 0 (two-sided t-test, p<0.05). **B**, Mean fixation-period activity of the 20 units that correlate best with either right or left choice, as a function of the number of trials relative to a choice switches from right to left or from left to right, on average across all MOs recordings. Analyses were performed in MATLAB R2023a (The MathWorks, Natick, MA, USA).

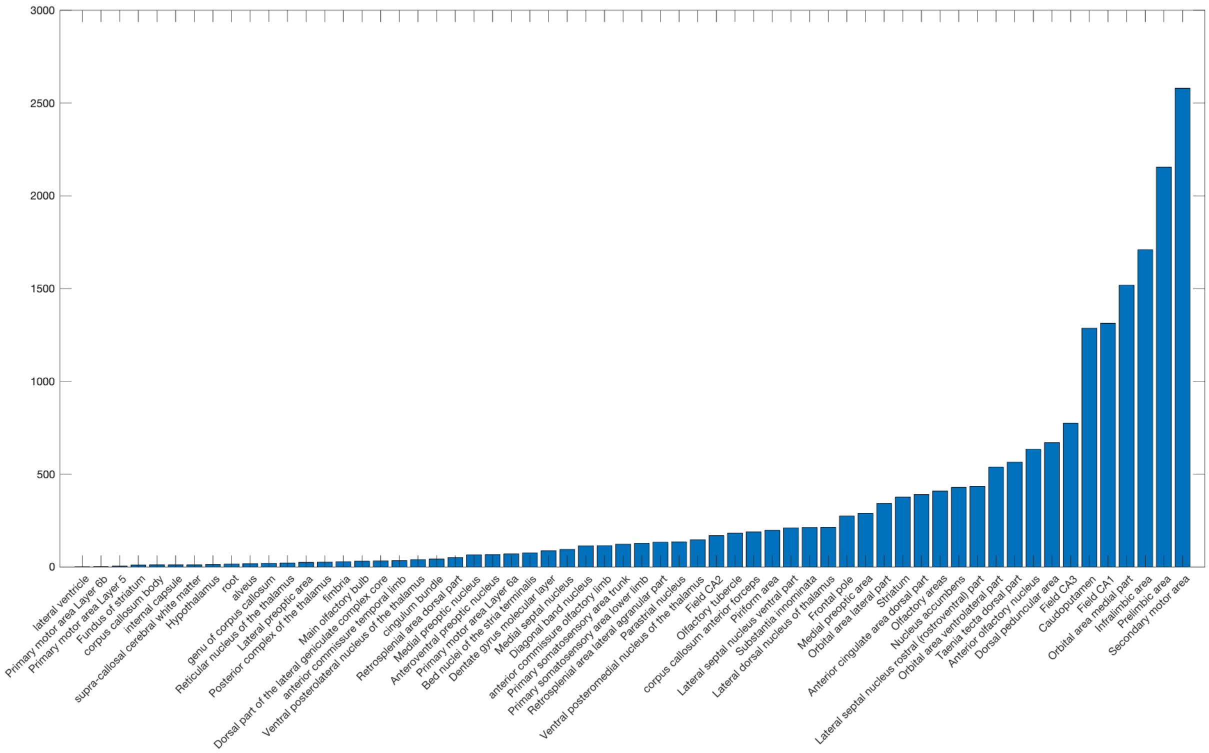

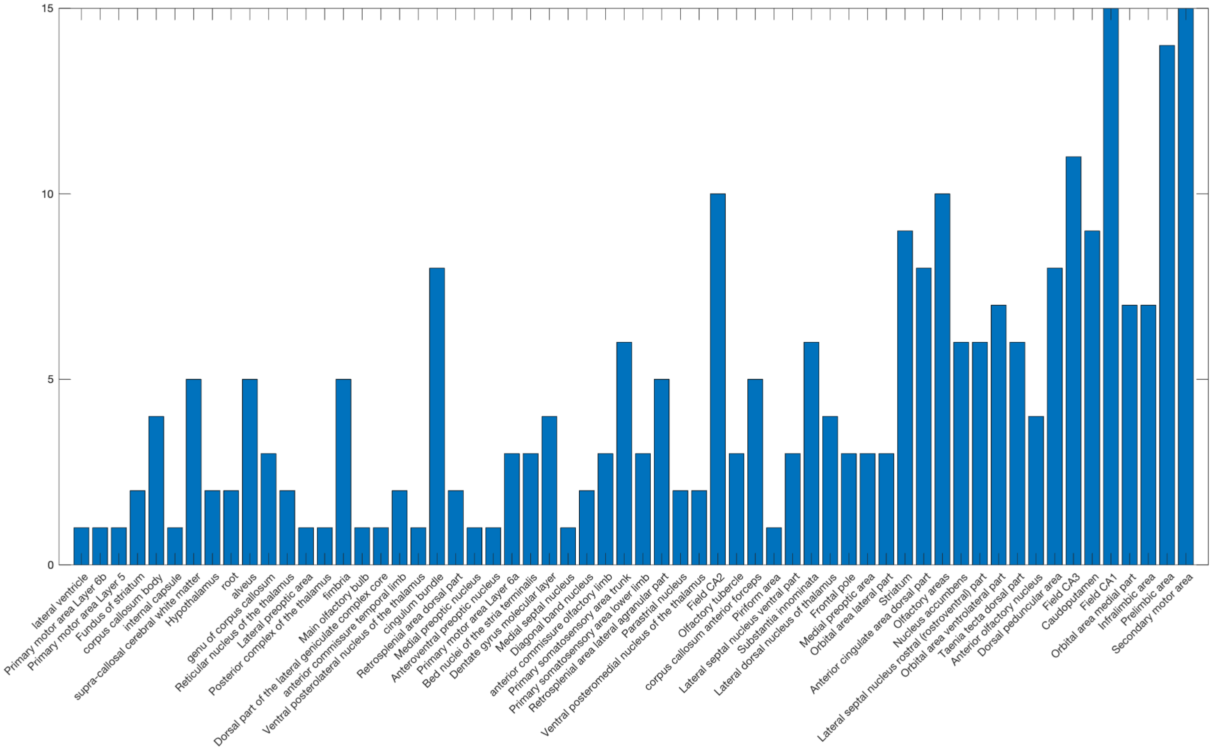

Supplementary figure 6. Top, number of units recorded in each area. Bottom, number of recording sessions per area. Analyses were performed in MATLAB R2023a (The MathWorks, Natick, MA, USA).

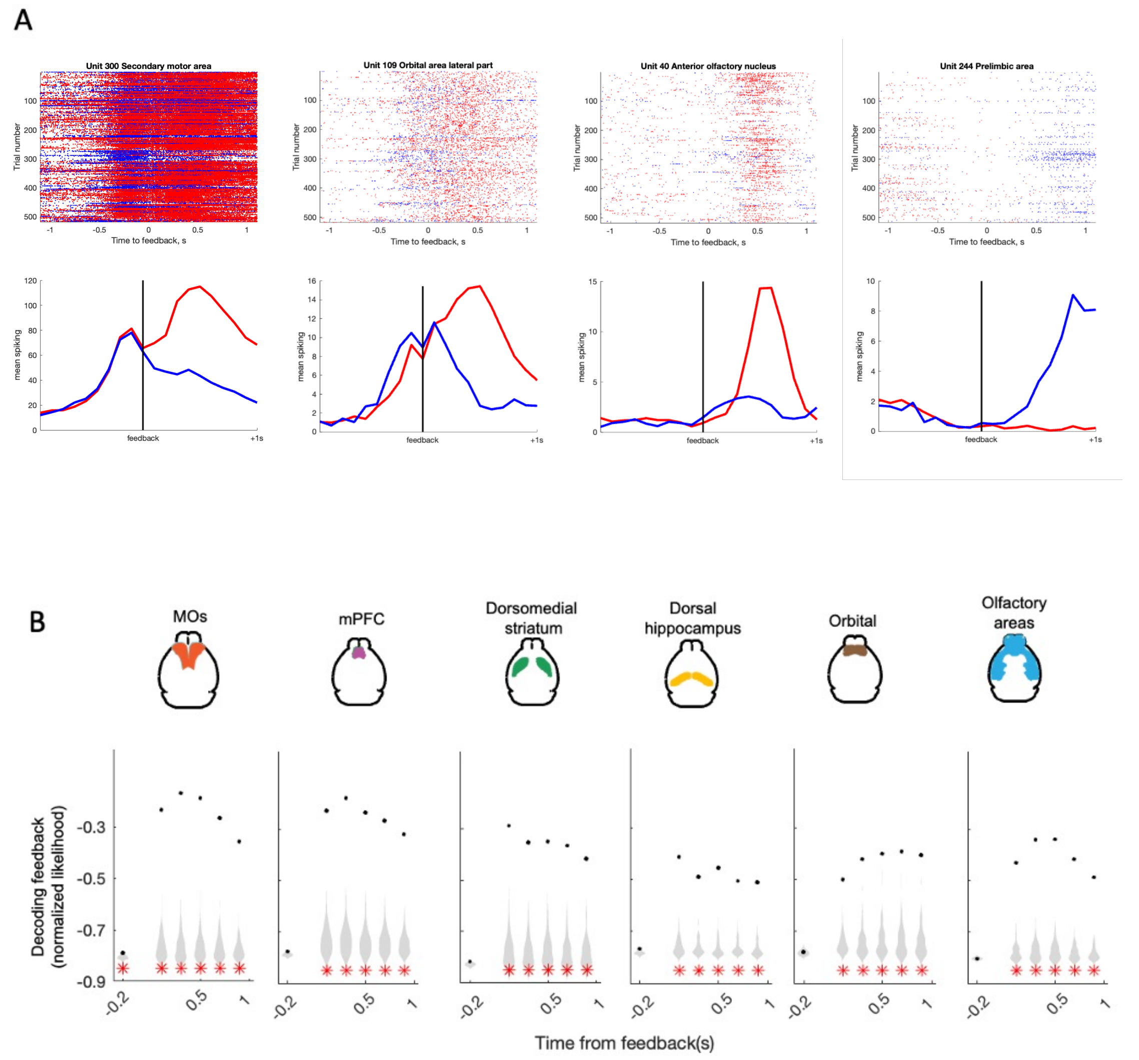

Supplementary figure 7. A, Activity of example units aligned to the feedback time, separated into rewarded (red) and not rewarded (blue) trials. Top: raster plots for four example units, trials ordered chronologically and colored by trial type. Bottom: peristimulus time histograms for the same units. Activity of each trials is aligned to the feedback time on that trial. **B**, Decoding of feedback from population activity. X-axis: time epoch; 0-200 ms before feedback, and subsequent 200 ms epochs from feedback onset. Black dots: mean prediction of actual value across sessions; gray violins, null distribution from session permutation. Red stars: p<0.01, two-sided session permutation test. Analyses were performed in MATLAB R2023a (The MathWorks, Natick, MA, USA).

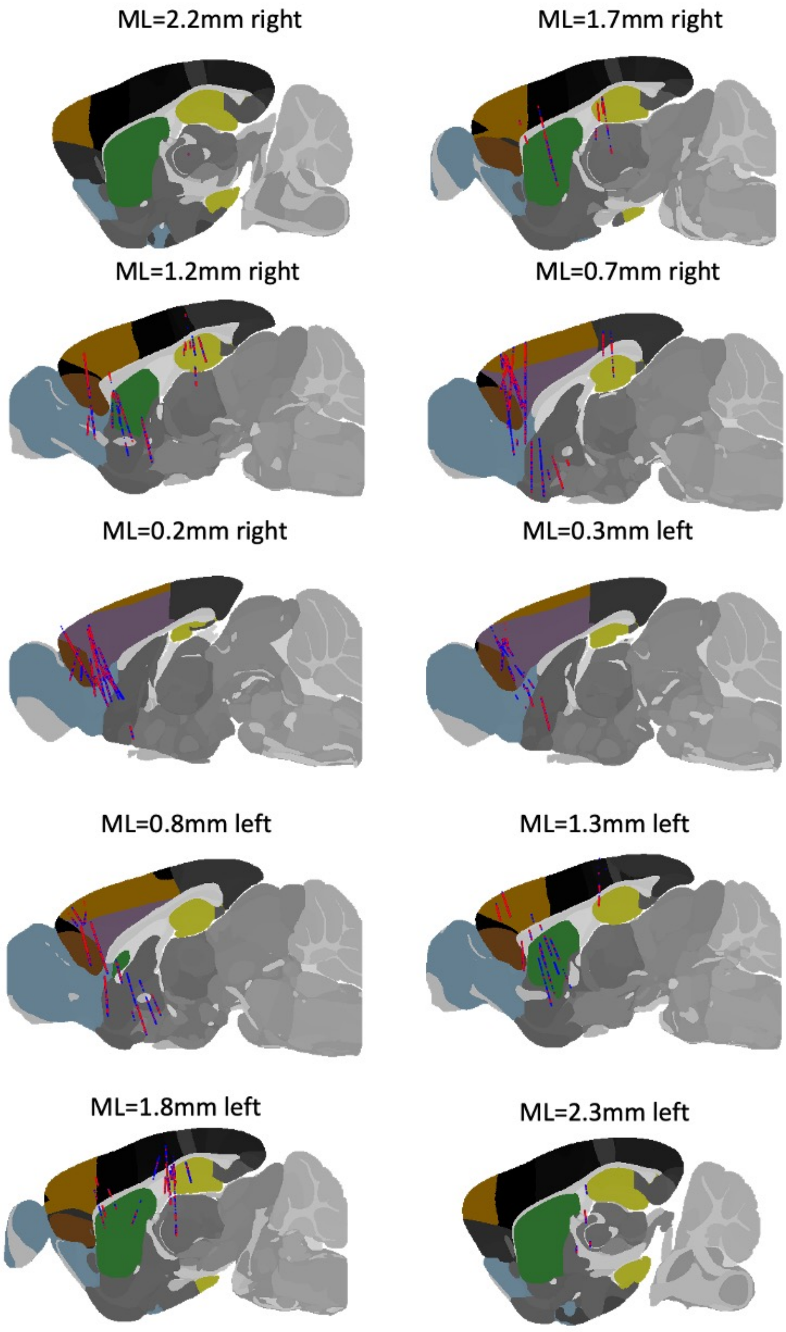

Supplementary figure 8. Spatial map of recording sites and choice correlation after the movement initiation. The maps show sagittal brain sections from the Allen Brain Atlas. The brain regions of interest are highlighted: MOs (orange), mPFC (lilac), orbital area (brown), dorsal hippocampus (yellow), dorso-medial striatum (green), olfactory areas (blue). Units that have significant (>0.15) correlation with choice after the movement has begun are marked red and the ones that do not are marked dark blue. Analyses were performed in MATLAB R2023a (The MathWorks, Natick, MA, USA). Images made using Allen Mouse Brain Atlas, mouse.brain-map.org and atlas.brain-map.org.

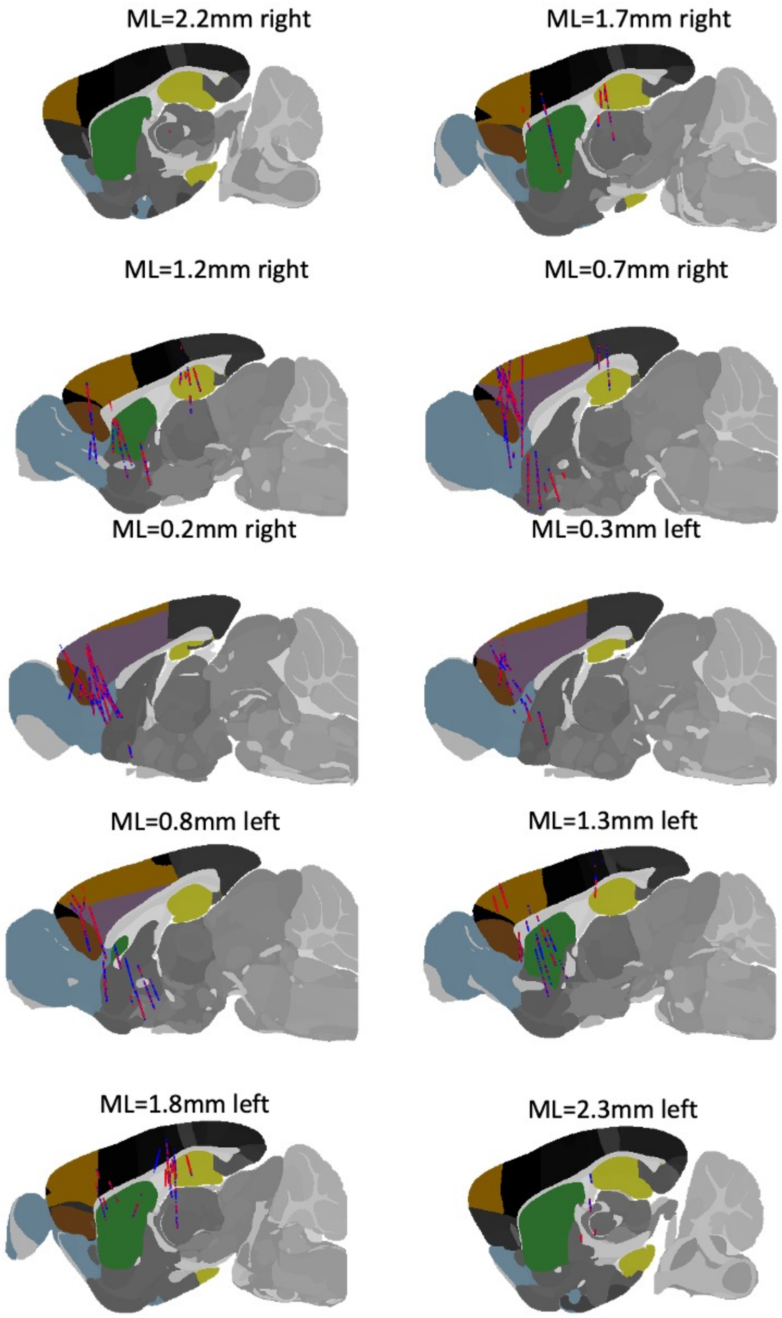

Supplementary figure 9. Spatial map of recording sites and feedback correlation after the feedback delivery. The maps show sagittal brain sections from the Allen Brain Atlas. The brain regions of interest are highlighted: MOs (orange), mPFC (lilac), orbital area (brown), dorsal hippocampus (yellow), dorso-medial striatum (green), olfactory areas (blue). Units that have significant (>0.15) correlation with choice after the movement has begun are marked red and the ones that do not are marked dark blue. Analyses were performed in MATLAB R2023a (The MathWorks, Natick, MA, USA). Images made using Allen Mouse Brain Atlas, mouse.brain-map.org and atlas.brain-map.org.

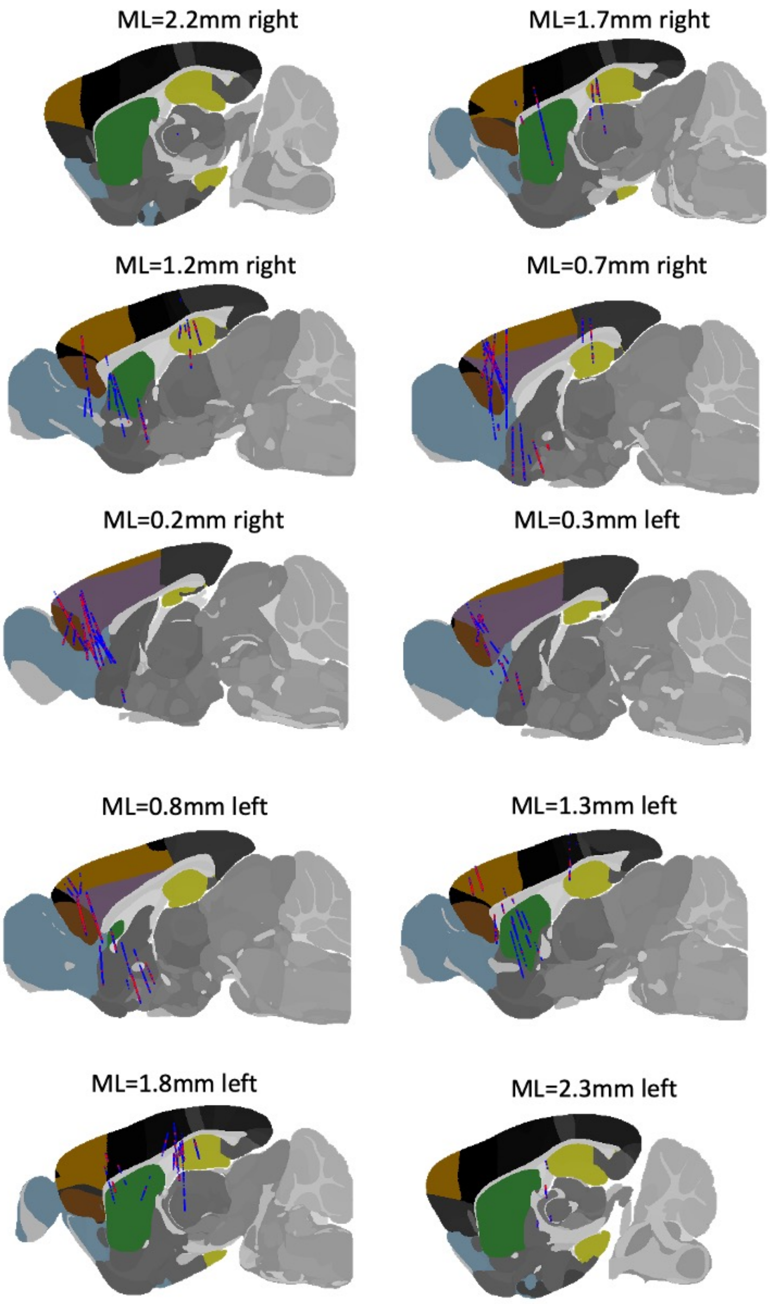

Supplementary figure 10. Spatial map of recording sites and choice correlation during the fixation period. The maps show sagittal brain sections from the Allen Brain Atlas. The brain regions of interest are highlighted: MOs (orange), mPFC (lilac), orbital area (brown), dorsal hippocampus (yellow), dorso-medial striatum (green), olfactory areas (blue). Units that have significant (>0.15) correlation with choice during the fixation period are marked red and the ones that do not are marked dark blue. Analyses were performed in MATLAB R2023a (The MathWorks, Natick, MA, USA). Images made using Allen Mouse Brain Atlas, mouse.brain-map.org and atlas.brain-map.org.

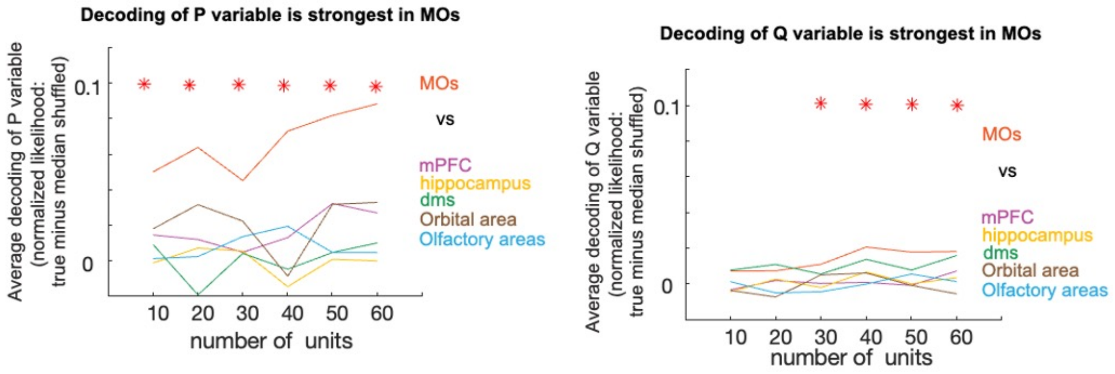

Supplementary figure 11. Decoding P and Q variables from fixation-period activity of equally-sized random subsamples of neurons from each region. X-axis: number of units in subsample; Y-axis, mean log-likelihood relative to permuted sessions. Red stars: MOs significantly different to other regions (p<.01; one-sided t-test). Analyses were performed in MATLAB R2023a (The MathWorks, Natick, MA, USA).

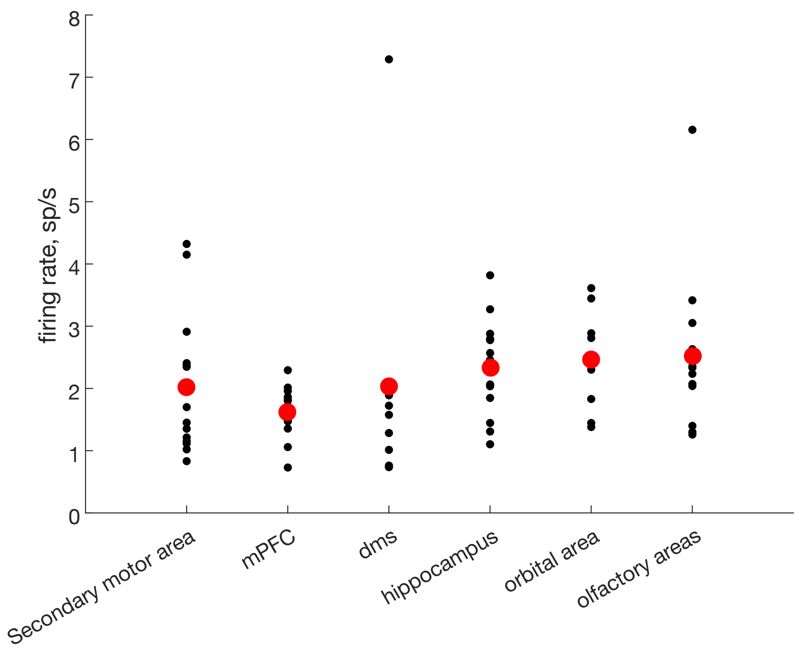

Supplementary figure 12. Firing rate during the fixation period. Each black dot represents mean firing rate of units in a specified region in a session containing at least 30 units in a specified area and red circles represent the mean across all sessions. No significant difference between MOs and any other area shown was detected (all p>0.05, two-sample t-test). Analyses were performed in MATLAB R2023a (The MathWorks, Natick, MA, USA).

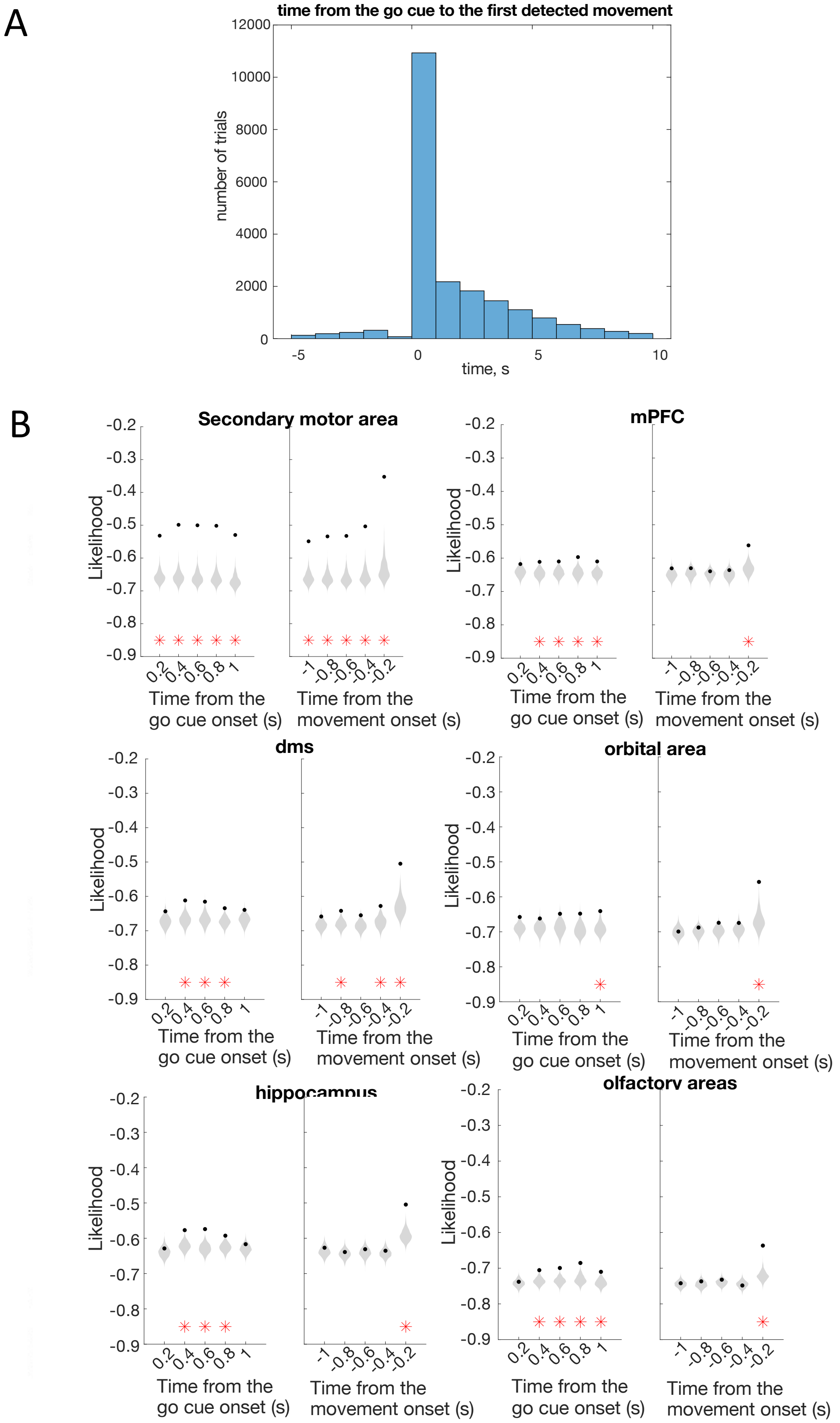

Supplementary figure 13. A, Bar graph showing the distribution of times between the first detected movement on a trial and the go cue time. **B**, Decoding of upcoming choice from population activity. Each column shows decoding of choice (logistic regression). For each region the decoding is done aligned to the go cue (left) and to the movement onset (right). X-axis: time epoch; 200 ms epochs after the go cue (left) or before movement onset (right). Black dots: mean prediction of actual value across sessions; gray violins, null distribution from session permutation. Red stars: p<0.01, two-sided session permutation test. Analyses were performed in MATLAB R2023a (The MathWorks, Natick, MA, USA).

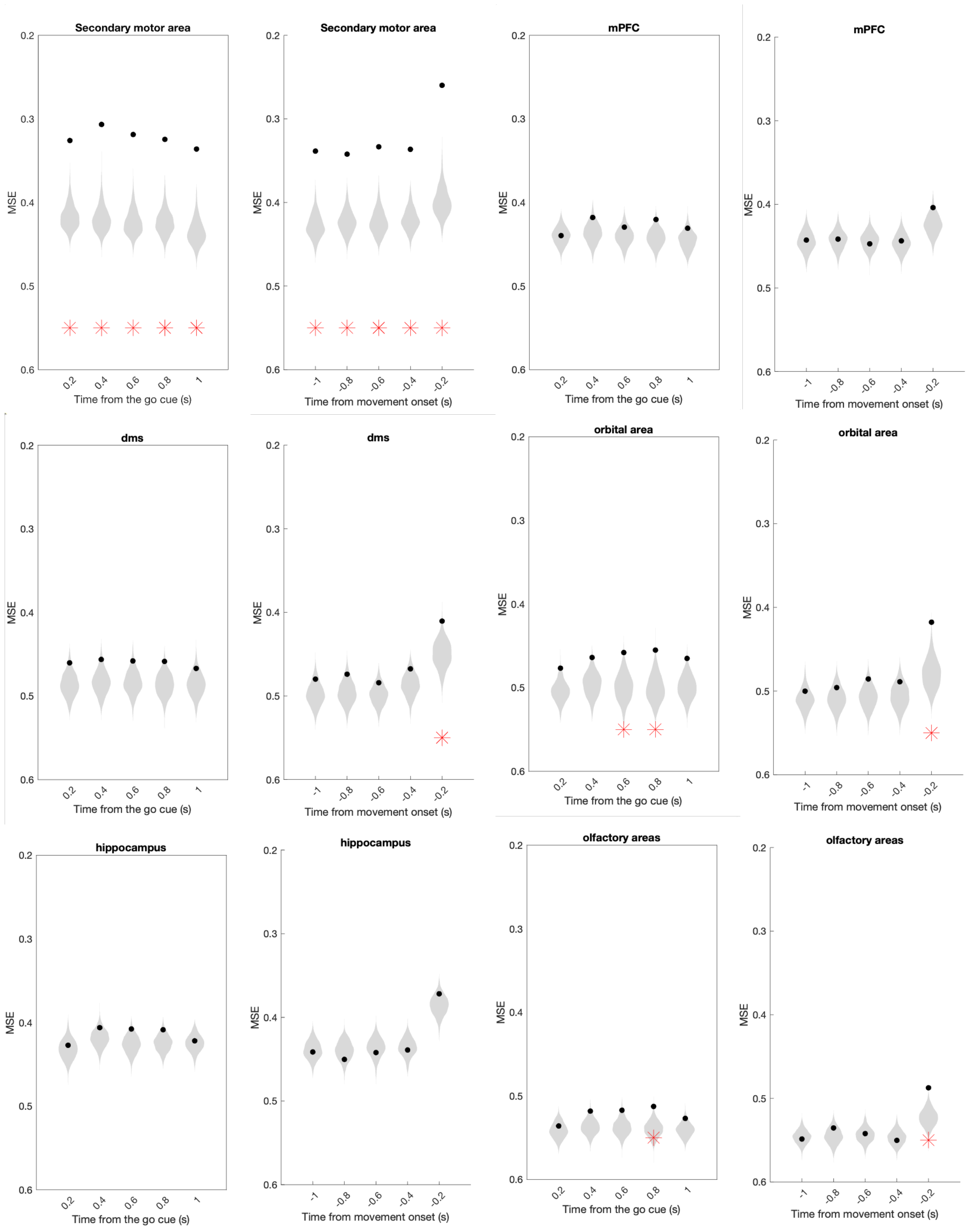

Supplementary figure 14. Decoding of P variable of the PR model from population activity. Each column shows decoding of P variable (linear regression). For each region the decoding is done aligned to the go cue (left) and to the movement onset (right). X-axis: time epoch; subsequent 200 ms epochs from go cue (left) or from movement onset (right). Black dots: mean prediction of actual value across sessions; gray violins, null distribution from session permutation. Red stars: $p<0.01$, two-sided session permutation test. Analyses were performed in MATLAB R2023a (The MathWorks, Natick, MA, USA).

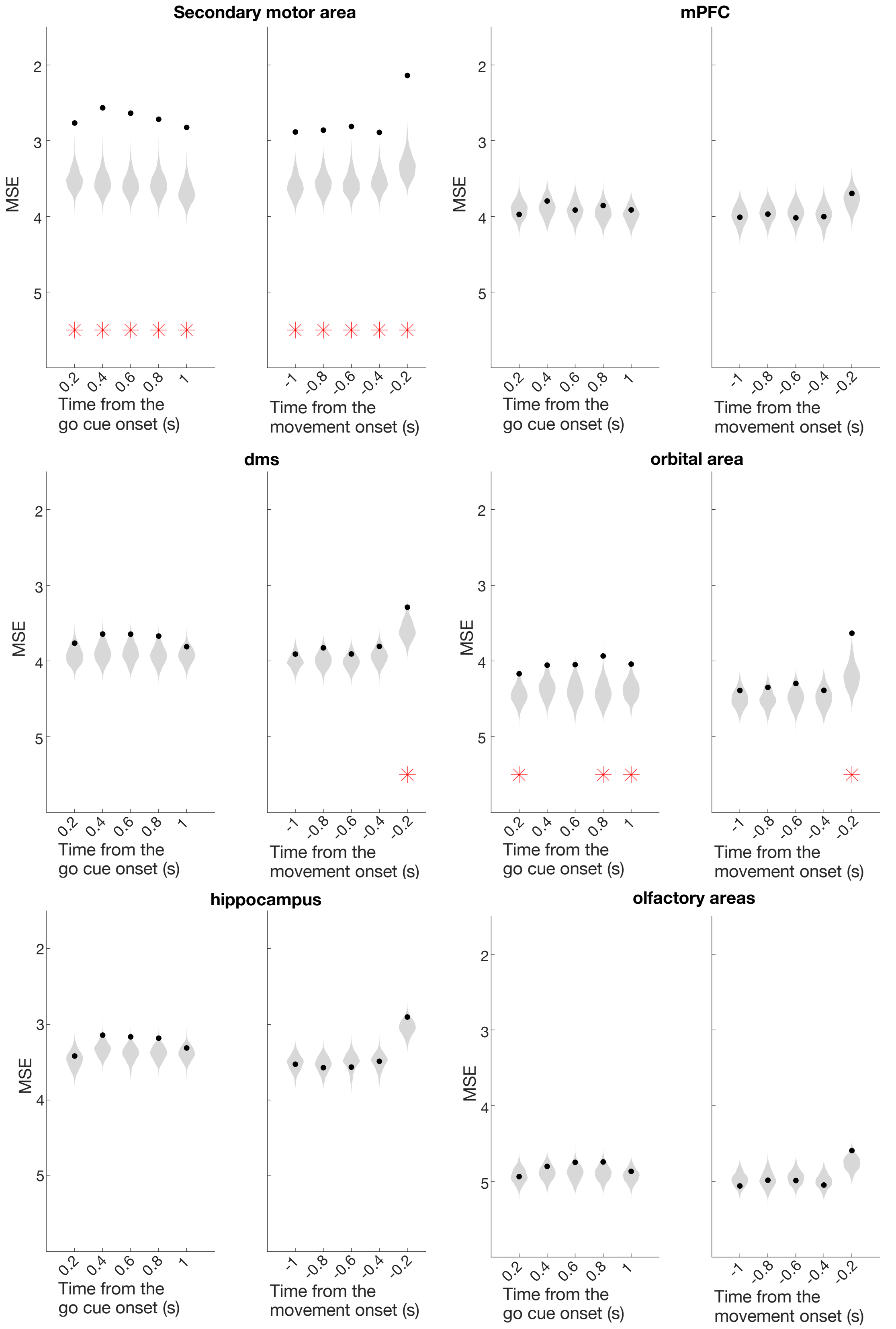

Supplementary figure 15. Decoding of P+R variable of the PR model from population activity. Each column shows decoding of P+R variable (linear regression). For each region the decoding is done aligned to the go cue (left) and to the movement onset (right). X-axis: time epoch; subsequent 200 ms epochs from go cue (left) or from movement onset (right). Black dots: mean prediction of actual value across sessions; gray violins, null distribution from session permutation. Red stars: $p<0.01$, two-sided session permutation test. Analyses were performed in MATLAB R2023a (The MathWorks, Natick, MA, USA).

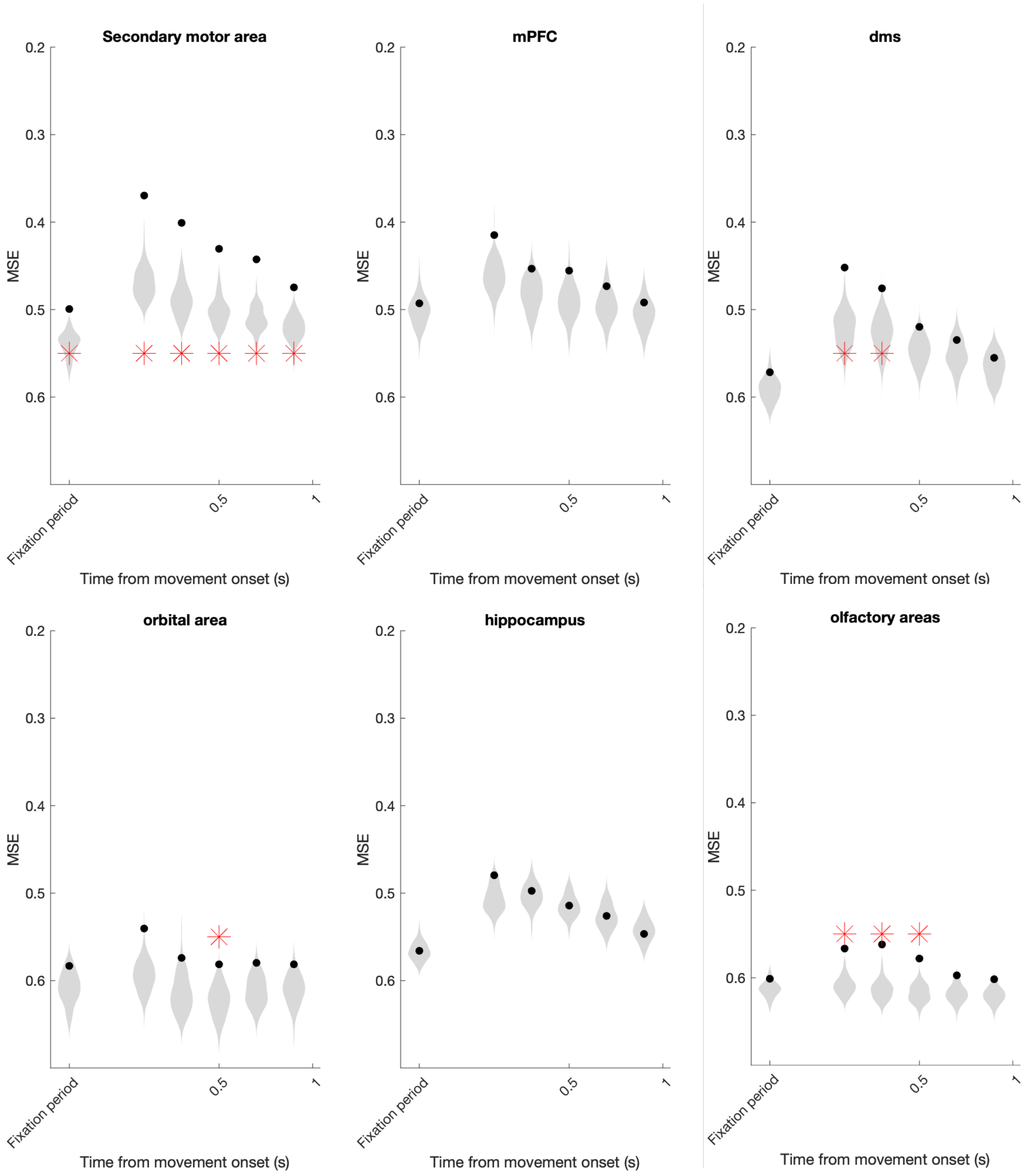

Supplementary figure 16. Decoding of perseveration variable of the PR model from population activity using LASSO regression. X-axis: time epoch; fixation period (0-200 ms before Go Cue), and subsequent 200 ms epochs from movement onset. Black dots: mean prediction of actual value across sessions; gray violins, null distribution from session permutation. Red stars: $p<0.01$, two-sided session permutation test. Analyses were performed in MATLAB R2023a (The MathWorks, Natick, MA, USA).

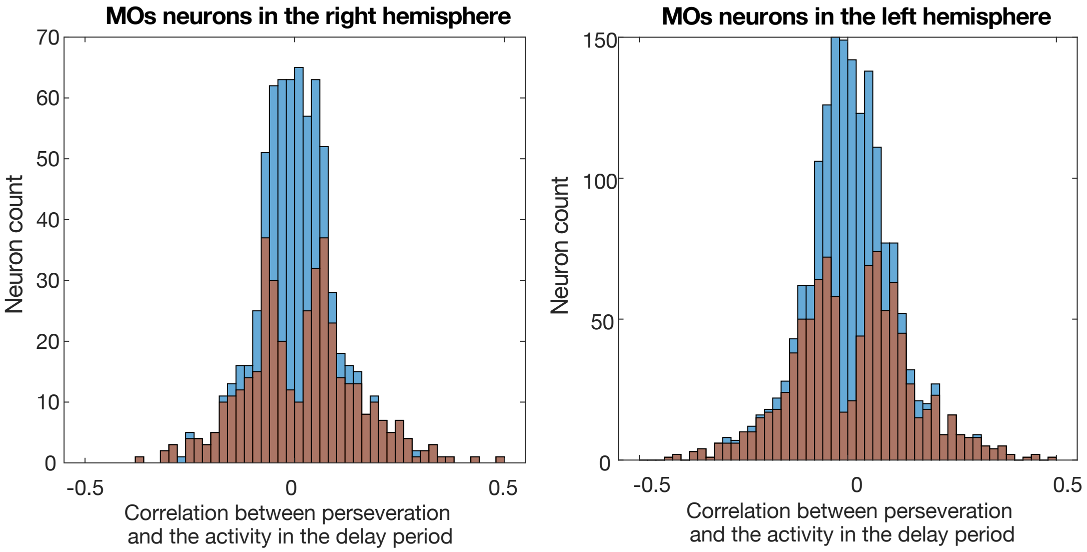

Supplementary figure 17. Left, correlation between activity of MOs clusters in the left hemisphere during the delay period and the perseveration variable. Blue, all units; orange, significant units according to session permutation test. Right, same for MOs units in the right hemisphere. Analyses were performed in MATLAB R2023a (The MathWorks, Natick, MA, USA).

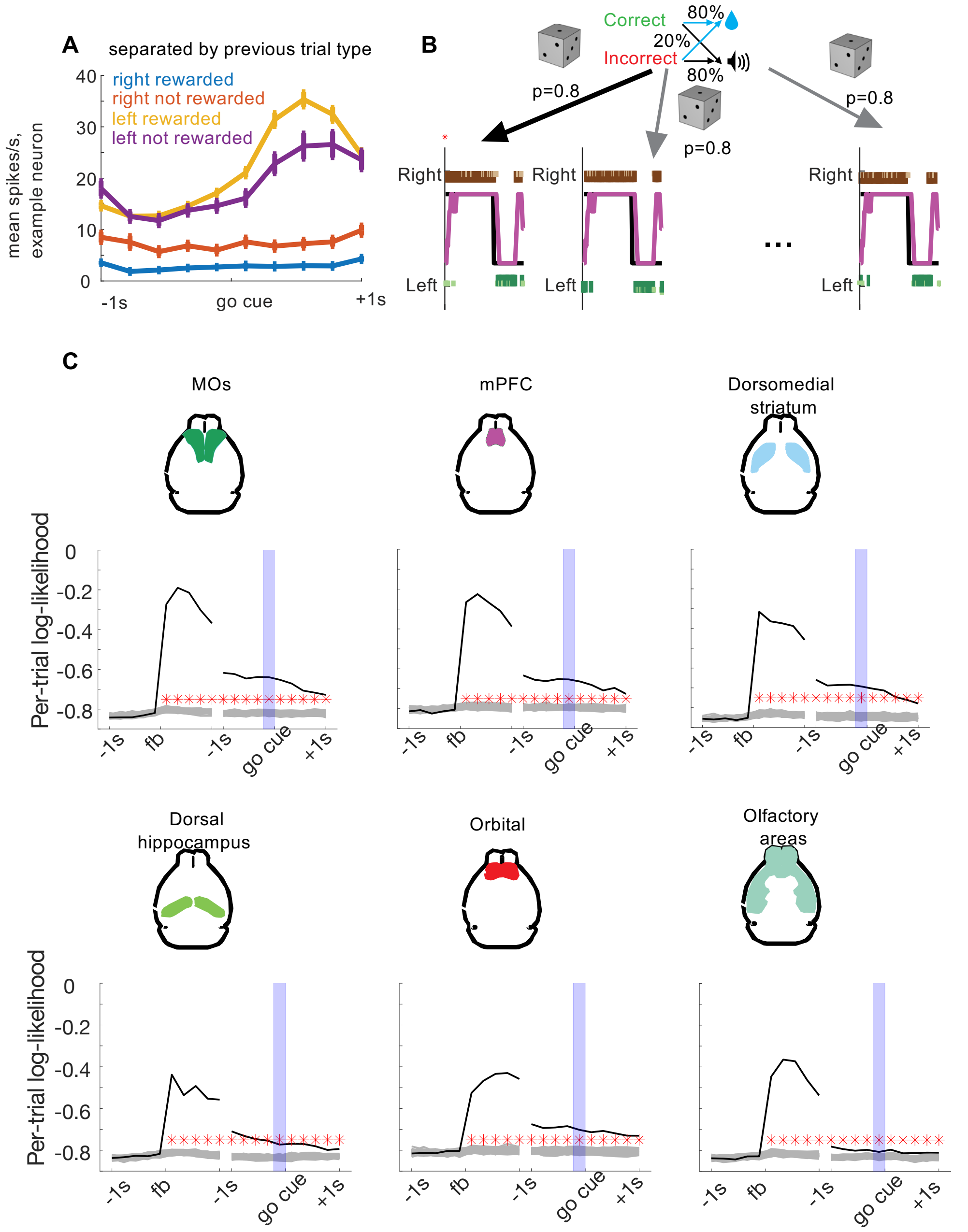

Supplementary figure 18. Causal effect of outcome (i.e. reward or white noise) on neural activity. **A**, Activity of an example neuron depends on the outcome of the previous trial (mean ± s.e across trials). **B**, To show a causal effect we took advantage of the fact that feedback delivery was randomized (80% or 20% reward probabilities for correct or incorrect choices). For each trial, we generated a null ensemble by resampling the outcome at random, with the same probabilities as in the original task, given animals' choices and correct responses. **C,** Accuracy of outcome prediction for the real session and the null ensemble. Black line shows log likelihood for predicting the actual outcome from neural population activity in a 200 ms time bin aligned to outcome time (left) or to the subsequent trial's go cue (right). Blue shading indicates the fixation period. Gray lines indicate null ensemble predicting randomly regenerated outcome sequences, drawn using the same behavior-dependent probabilities as the actual sequence. Red stars indicate time bins in which the likelihood for predicting the actual choice exceeds 99% of the null ensemble. Note that even if the neural activity correlates with a prediction of upcoming reward magnitude, this analysis could not show a causal effect of outcome on activity before outcome delivery: because the null ensemble was randomly generated with the same probabilities as the actual outcome, there is no way that neural activity could predict the actual outcome better than null, even if it could predict whether the animal would choose the correct or incorrect side. The dice in panel B were produced in MATLAB R2023a (The MathWorks, Natick, MA, USA); analysis performed in MATLAB R2023a (The MathWorks, Natick, MA, USA).

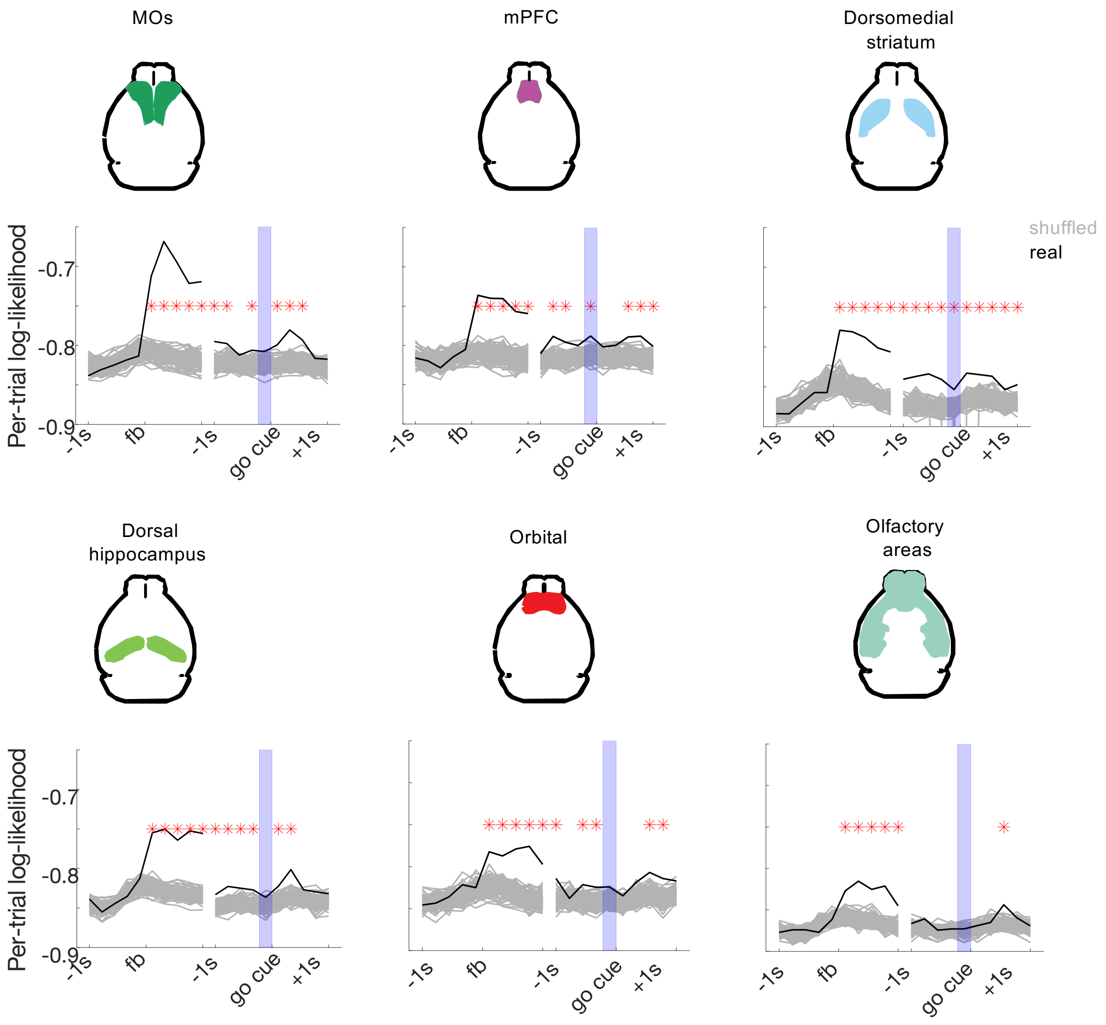

Supplementary figure 19. Causal effect of choice-feedback interaction on neural population activity, assessed by the same method as Figure 6. The analysis proceeds exactly as in Supp Fig. 6, but predicting the choice-feedback interaction rather than just the outcome value. Black lines indicate prediction of actual value, gray lines indicate the null ensemble obtained by resampling outcome values with the probabilities determined by the mouses actual choices, then recomputing the choice-outcome interaction. Blue rectangle, fixation period. Red stars indicate time bins in which the likelihood for predicting the actual choice-feedback interaction exceeds 99% of the null ensemble. Analyses were performed in MATLAB R2023a (The MathWorks, Natick, MA, USA).

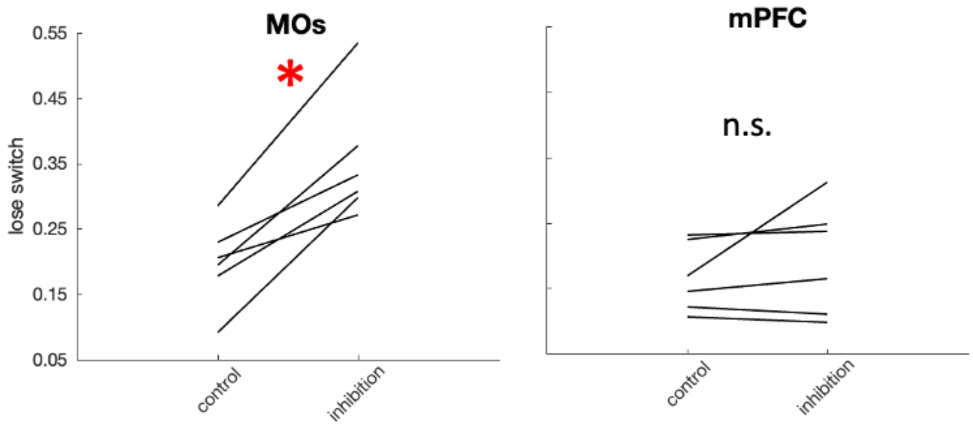

Supplementary figure 20. Probability of switching choice after no reward in no laser (control) and laser (inhibition) condition in MOs (left) and mPFC (right). Laser stimulation delivered upon the go cue. n=6 mice; paired t-test (left, $p = 0.002$; right, $p = 0.28$). Analyses were performed in MATLAB R2023a (The MathWorks, Natick, MA, USA).

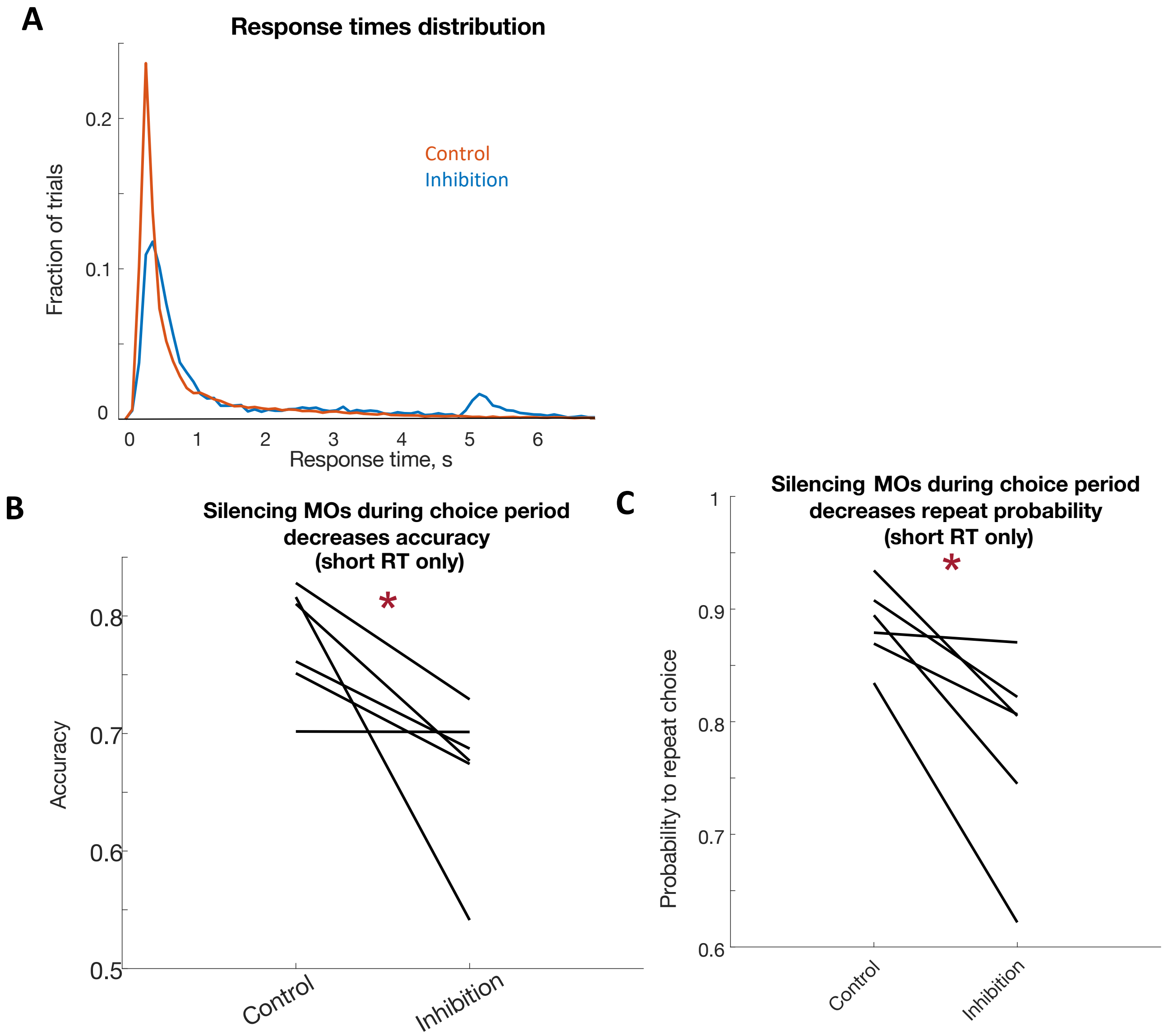

Supplementary figure 21. A, Distribution of response times in control trials (orange) and trials with inhibition in MOs during choice period (blue). **B,** Same as figure 6D but only including trials with response times less than the duration of opto stimulation. The star indicated that the is statistically significant difference between the control and inhibition conditions (paired t-test, p=0.03). **C,** Same as figure 6E but only including trials with response times less than the duration of opto stimulation. The star indicates that there is a significant difference between the control and inhibition conditions (paired t-test, p=0.01). Analyses were performed in MATLAB R2023a (The MathWorks, Natick, MA, USA).

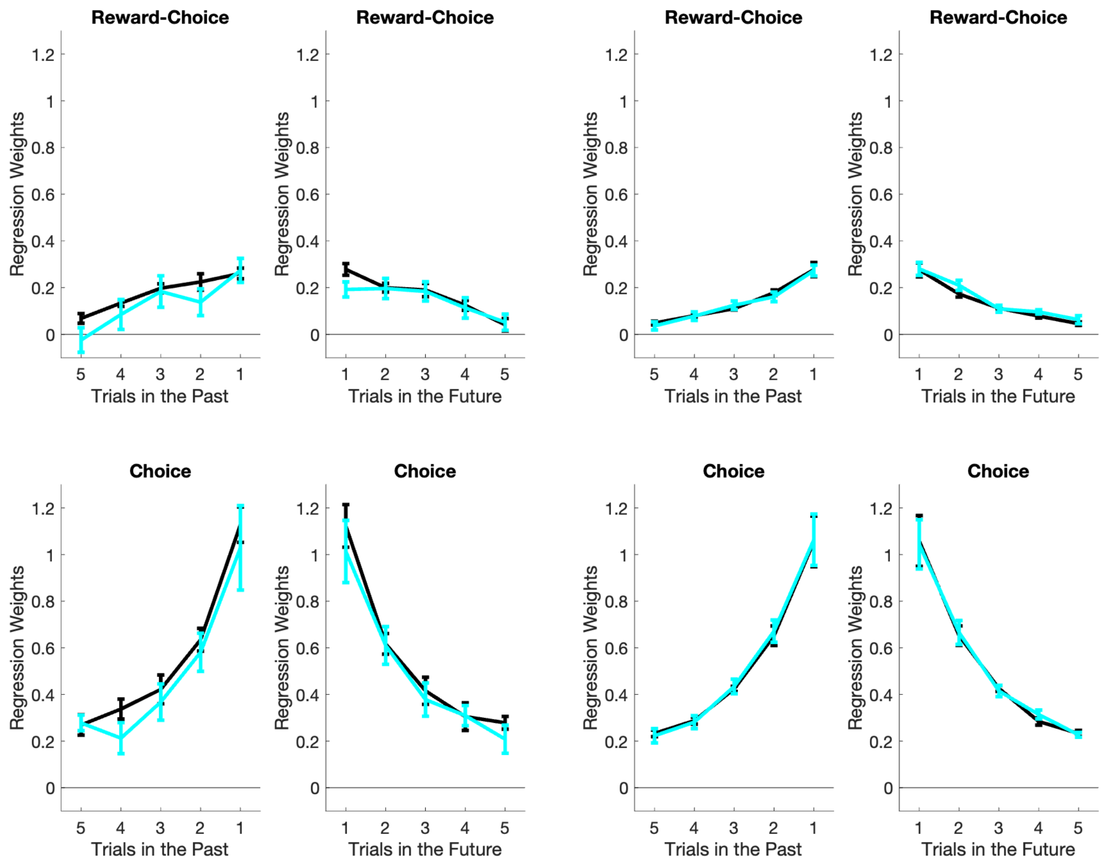

Supplementary figure 22. mPFC activity is not required for perseveration. Left 4 panels, Trial history regression weights for predicting mouse choice using past choices (choice weights), and choice-reward interaction (reward-choice weights), for laser (cyan) and no laser (black) conditions. Left column shows weights from trials N-n to trial N, where inactivation was delivered to mPFC on trial N; right column shows weights from trial N to trial N+n, where inactivation was delivered to mPFC on trial N. Right 4 panels show the same but for simulated data from PR model with no effect of optogenetic manipulation. (n = 6 mice; mean ± s.e.m.). Analyses were performed in MATLAB R2023a (The MathWorks, Natick, MA, USA).

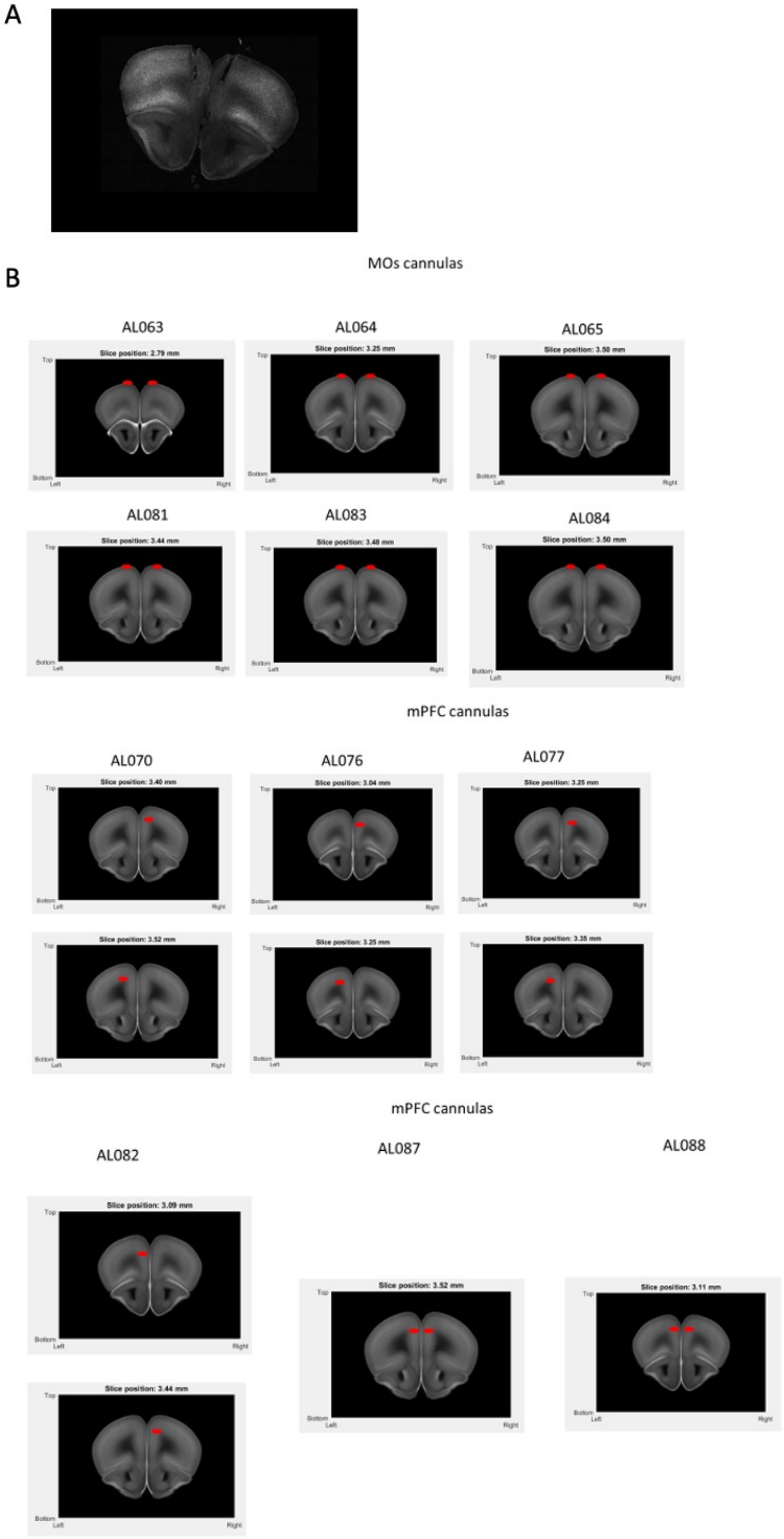

Supplementary figure 23. A, Example slice containing two cannula tracks (animal AL088). **B**, Estimated positions of the bottom of each cannula for optogenetic experiments (see methods). Images made using Allen Mouse Brain Atlas, mouse.brain-map.org and atlas.brain-map.org.
