# Supplementary material for: Dorsal prefrontal cortex drives perseverative behavior in mice
Source: Nat Commun. 2026 Apr 25;17:5715. doi: 10.1038/s41467-026-71664-w (PMC13324276; doi:10.1038/s41467-026-71664-w)
Supplement: Supplementary file 3 — Transparent Peer Review file [file 41467_2026_71664_MOESM3_ESM.pdf]

nature portfolio

Peer Review File

Dorsal prefrontal cortex drives perseverative behavior in mice

Corresponding Author: Professor Kenneth Harris

This file contains all reviewer reports in order by version, followed by all author rebuttals in order by version.

Version 0:

Reviewer comments:

Reviewer #1

(Remarks to the Author)
In this study, the authors examined the neural circuits pf perseverative behaviour in Mice.
Using a probabilistic reversal task in head-fixed animals, the authors first examined the computational rules for performing in this task. They therefore compared the performance of 13 models using cross-validated likelihood and found that 5 models captured similar aspects of the mice behaviour. The authors chose the simplest one (so called perseverative/reward model, Miller, 2011) in order to fit the model with neural data.
Using Neuropixels probes, the authors then record neuronal activity in a set of forebrain structures, including the secondary motor area (MOs, available data for 15 sessions), the mPFC (available data for 14 sessions), the OFC (available data for 11 sessions), olfactory regions (available data for 15 sessions) and dorsal hippocampus (available data for 15 sessions). Using a set of sophisticated analysis, they found that the neural activity in MOs was the most predictive of the upcoming choice. Critically, they found that such an activity predicts the perseverative element of computation, but not the reward seeking element one. Finally, using optoinhibition, they found that inhibiting the MOs at the time of choice decreased perseverative behaviour.
Taken as a whole, this is a very well-written paper, which makes a very interesting contribution to the field. I have a few comments which are listed below.

1. The first comment is about the training
The authors state that the animals became expert in the task following 4 to 12 weeks of training. Could the authors provide some details for each mice about the acquisition of the task. It could also be very useful to be provided with a description of a possible relationship between the speed of learning and the amount of perserverative behaviour. Therefore, given that one might expect the animals strategy to gradually change with training, the authors should therefore provide evidence of such changes by providing training data.

2. The second comment is about the targeted cortical areas and anatomical boundaries.
Previous research has demonstrated that choice in such task is regulated by different regions of the prefrontal cortex. Therefore, the authors should make clearer their rationale of exploring the role of the various brain regions recorded, in particular the DH and the olfactory regions. Also, the anatomical boundaries of the recorded areas should be better described given that important anatomical and functional dissociations have been described at the level of both the MOs and the mPFC. Importantly, current research has provided convincing evidence for a role of the OFC in adapting to change in contingencies in such a probabilistic task. Therefore, the lack of neural modulation in this brain region should be better discussed in relation to the well known role of the OFC in the Exploration/Exploitation trade-off and also win-stay/lose-shift behaviours.

Reviewer #2

(Remarks to the Author)
This study suggests a role for the secondary motor cortex (MOs) in a dynamic reward-learning task as a driver of perseverative choice behavior. Prior to movement, MOs represented the upcoming choice and a perseverative variable which was estimated from a behavioral model. Optogenetic inactivation during the decision period, achieved by stimulating interneurons in MOs, reduced repeated choice behavior, thereby impairing value-based decision-making. A similar

experiment in the prefrontal cortex (PFC), conducted during the outcome period and in subsequent trials, slowed learning of switching reward probabilities, with a significantly larger effect size compared to MOs. These results highlight distinct roles for MOs and PFC at different stages of value-based decision-making.
This study examined choice behavior by analyzing two key variables: choice-related (perseverative, P) and value-related (reward-seeking, R) factors, proposing another view for interpreting behavior in a dynamic reward-learning task. Plus, although previous rodent studies (Kim et al., 2009; Sul et al., 2010; Sul et al., 2011; Lee et al., 2012; Bari et al., 2019; Hattori et al., 2019) demonstrated that most of the regions (excluding olfactory areas) recorded in this study signaled choice and/or value variables, such as action- and chosen-value, this study examined the neural representation of perseverative and reward-seeking variables. Additionally, while earlier studies showed that lesions in MOs in rats (Sul et al., 2011) and pharmacological inactivation of PFC in mice (Bari et al., 2019) impaired value-based choice behavior, this study investigated the specific stages within each trial and across trials that depend on the involvement of these regions. However, to fully integrate and build upon the findings of this study, several points require further clarification.

1) Decoding of choice, perseverative variable (P) and reward-seeking variable (R), relate to Figure 4A
The fixation period may warrant further focus as it excludes motor responses and allows investigation into the aspect of planning. In the optogenetic experiment, since the laser was applied after the go cue (and not during the fixation period), it is important to examine neural representations during that period. While decoding results were presented after movement onset, have you examined the decoding results for the period between the fixation offset and movement onset? Given the potential variability in the length of this period, focusing on data from around the fixation period, movement onset, or presenting analyses similar to those in Supplementary Figures 6 and 7 could provide additional insights.

2) Neural representation of decision variable (P+R), related to Figure 5C
Similarly to the above point, have you examined the temporal pattern of neural representation for P+R (which may serve as the decision variable in this study) as shown in Supplementary Figures 6 and 7? The optogenetic experiment in MOs during the decision period decreased the weight of previous reward-choice interactions (Figure 6I), yet the reward-seeking variable does not appear to be represented in MOs (Figure 4A). Additionally, since the laser was not applied during the fixation period, it would be important to analyze neural representation after the matching period.

3) Result of optogenetic experiment in PFC, related to Figure 6
Similar to MOs, how should we interpret the results of the optogenetic experiments in the PFC in relation to its neural representation during this task? During the outcome period, the reward-seeking variable is likely important for updating variables relevant to the next choice. However, the level of its representation appears to be stronger in MOs compared to PFC (Supplementary figure 7). Alternatively, if it is possible to examine updating-related variables in the model used in this study, such as reward prediction error or updated chosen-value from a Q-learning model that dynamically changes during transitions, could these variables be more prominently represented in PFC than in MOs?
In this context, considering or comparing the results of Bari et al. (2019), which demonstrated stable representations of decision variables from the Q-learning model in the PFC that are critical for value-based choice, with findings of this study could provide additional insights. Such comparisons might further highlight the distinct contributions of this study and provide more comprehensive information to readers.

4) Comparison with previous studies, related to Discussion (line 379 – 389)
In Sul et al. (2011), lesions of the MOs increased lose-stay behavior but decreased the inverse temperature parameter in the Q-learning model, suggesting that switch or stay behavior may depend on previous choices and rewards. In this study, have you examined lose-switch behavior and model parameters, particularly those related to perseveration?
Additionally, experimental details such as precise reward schedules and head fixation vary across this study and earlier works (Sul et al., 2011; Hattori et al., 2019). In particular, the ratio of reward probabilities between the two rewarding sides is larger in this study compared to previous ones. These methodological differences may contribute to variations in observed choice behavior.

5) Minor descriptive suggestions
Regarding Figure 1F (lines 88–89), have you tested for statistical differences between the weights of choice and those of reward-choice interactions? Additionally, interpretation or discussion related to Supplementary Figure 4B appears to be missing. Lastly, Supplementary Figure 9 should be referenced in the corresponding descriptions for clarity and completeness.

Reviewer #3

(Remarks to the Author)
Lebedeva et al measured activity in multiple cortical brain regions during a task shown to produce perseverative behavior in mice, then optically inhibited selected brain regions to determine if this activity was causally linked to perseverative behavior. They observed that dorsal PFC activity predicts both perseverative behavior and response times, and inactivation of this region reduced perseveration. The technical aspects of this paper are excellent, and I admired the rigorous testing of different computational behavior models as well as the concise summaries of these approaches in the methods. I have some relatively minor concerns about interpretation, use of male/female mice, and the unclear criterion used for task comprehension, which are detailed below.

1. Further discussion of the cause of MO driving of “perseverative behavior” is warranted, as perseverative choice could reflect different scenarios. For example, perseveration may be a result of non-outcome driven, stimulus-response/habitual

behavior. Alternatively, perseveration could be use of an exploitation over exploration strategy, with MO inactivation shifting mice toward increased exploration. Please discuss briefly in intro or discussion.
2. Unless I missed it, there is no mention of sex in this report. Please add this information, and provide rationale if only one sex was chosen.
3. The authors report that MO units encode direction of upcoming choice – can you provide evidence that this is not solely a spatial left vs. right signal, and instead has some link to goal-directed actions/outcome expectancy?
4. In “Behavior Training”, it is unclear what criterion was used to determine that mice were trained in the task. Please provide more details – this is very important, as choice behavior in some subjects might reflect inability to discriminate between options rather than perseverative choice.
5. In the “optimistic Q learning” section of methods, the term “negative reinforcement” is used incorrectly – in the instrumental literature, this term refers to performing an action to remove an aversive stimulus (rather than an action no longer being reinforced as in this experiment).
6. When using optogenetics, it is good practice to include an optical stimulation condition during the ITI to control for non-specific effects (i.e. effects of optical inhibition might not be related to manipulation of event-specific activity, but instead caused by longer-lasting effects of inhibition on overall activity). Please add a brief explanation for why this is not a concern (I think the diverging effects of MO inhibition during different behavioral events should be sufficient to explain this).

Reviewer #4

(Remarks to the Author)
I reviewed the ms by Lebedeva et al. together with two lab members working on very related topics and methodology.
The authors investigate the behavior and brain activity of mice engaged in a probabilistic two-choice reversal learning task using a combination of behavioral modeling, electrophysiology, and optogenetics. The study states to address which brain regions carry neural activity that correlates with, and has a causal role in, perseverative learning and decision-making. Whether perseveration is supported by a similar set of brain regions and internal cognitive variables as reward-guided learning and decision-making is also addressed.
A model was fit to the behavioral data, deriving behavioral variables representing perseverance (repeating previous choices) and reward-based learning.
Findings:
behavioral analysis: Mice persevere more than would be optimal for reward-maximization.
neuronal activity: Measuring neuronal activity in several brain regions extracellularly using Neuropixels, the authors report that choice and perseverance can be best decoded from (correlates best with) activity in the MOs.
optogenetic manipulation: Driving PV+ neurons in the MOs during the choice period reduced task performance and perseverance. Stimulating PV+ neurons in the “mPFC” after reward delivery impaired reward-based learning and caused slower adaptation to rule changes.
The authors conclude that MOs "encodes" the perseverative behavioral variable (or "drives preservation") rather than reward-optimization.

REVIEW SUMMARY
Strengths: Promising approach and data
In combining behavioral modeling, electrophysiology, and optogenetics the study employs a promising approach. The focus on perseverance, a relatively underexplored behavioral variable, adds novelty and the dataset appears robust, enabling a potentially nuanced exploration of the relationship between neuronal activity and decision behavior. The thorough behavioral analyses are a strength and provide important and interesting insights into the law of exercise and perseverative behavior.

Weakness: scientific rationale and data presentation
The study is quite straightforward - one behavior and a few, complementary and relevant methods. However, the presentation of the study does not reflect this and the rationale behind what is analyzed and presented versus not is enigmatic. The presentation gives the sense of an agenda different from a wish to truly investigate neuronal underpinnings of perseverative learning and decision-making, reducing the potential impact of the study.

The recorded neuronal activity is arguably the most important part of the study and a good amount of work should have gone into collecting this data (>19,000 units in the final dataset, however, the rationale behind the choice of brain subregions is unclear). It is therefore a mystery why not more effort and focus are put into analysis and presentation of this dataset. Concerning the actual experiments, we find the lack of spatial precision in analyses and presentation troublesome, hindering true insight into the circuits of perseverance. Beyond the selection of what is presented, we unfortunately find shortcomings in the way data and methods are presented and discussed (see further below).

Our overall view: the study addresses an interesting topic with a potentially suitable approach. However, substantial issues in manuscript organization, data selection and presentation, and explanation of methodology hinder a judgment of the study’s scientific quality and reproducibility. These issues should be addressed before comments on specific revisions can be made.

Major points outlining what we find problematic

Manuscript organization
We find that the data representation in the manuscript is unnecessarily complicated and puzzling. Figure legends, while

being wordy, offer little aid for comprehension. The ms manages to be shallow and complicated at the same time, which we think stems from the disconnect between what is said to be the focus of the study to what is actually analyzed/presented. There is a disproportion between what is given space versus not, with the lack of presentation of central data compromising the value of the study. While we find the behavioral analysis powerful, we find the neuronal activity to be neglected. Much of the analysis focuses on small subsets of neurons, with panels displaying example neurons, obscuring a view of the activity involved in perseverative learning and decision-making. This also gives a troubling flair of cherry-picking and contributes to the questioning of the study's scientific foundation and goal.

The Results section includes information that is better suited to the Methods section (e.g., detailed explanations of nonsense correlations, comparisons of similar behavioral models, Fig. 1G). Conversely, aspects that are arguably central to understanding and evaluation of the scientific content are buried in the Methods section, e.g. what the authors mean by "neural activity".

Data validity

Anatomical precision

First, the scientific rationale behind what anatomical territory is recorded is unclear - the selection appears rather random. Furthermore, mapping of the anatomical position of Neuropixels recording sites is standard, e.g., using SHARP-Track, giving insight into 3D anatomical locations, layers etc. In the current study, anatomy is overall neglected, both in analysis and the interpretation of the data. The MOs encompasses a very vast anatomical territory, and the authors make claims about MOs as a whole while in reality likely sampling and analyzing a small sub-territory - we find this to be a serious problem. Overall, it is unclear where specifically the activity originates, as the provided information and figures are very superficial. Several PFC subregions are lumped into mPFC, without a clear scientific motivation. Lumping of subregions (into PFC/HPC/ORB/olfactory etc.) cannot be justified given the current state of the art and does not reflect the frontline of the field. Beyond the unnecessary inaccuracy lumping entails, it also calls some of the analyses into question. We e.g. wonder how it affects the selection of random neurons. On a more basic level, we wonder how the neurons in the whole dataset correlate with the behavioral variable, where exactly are the highest correlations found, how correlations vary across anatomical 3D space etc.?

In terms of anatomy, we find that the authors do a disservice to both the study and the research question by treating brain regions as if they held entirely separate functions. This approach might be particularly misleading for the PFC, as extensive data show shared connectivity between its regions, with gradual rather than abrupt shifts in connectivity (see eg Gao et al., 2022, DOI: 10.1038/s41593-022-01041-5). For example, some areas of the MOs may exhibit more similar connectivity to adjacent medial mPFC regions than to distant parts of the MOs - this would have implications on functon(s). A more unbiased, data-driven approach to analyzing the recorded activity and its correlation to behavioral variables would significantly strengthen the study.

Neuronal activity: definition and selection process

The selection process for which units to include (only considering the 20 single units whose "activity" is most correlated to a behavioral variable) is not justified. Furthermore, it appears 20 units are selected based on their individually strong correlations with e.g. the behavioral variable. However, 20 units that are by themselves not so correlated with the behavioral variable could potentially TOGETHER predict the behavioral variable better. It is to be expected that the units that are individually very correlated to the behavioral variable are also very much correlated among themselves (so from an information perspective, the selection process is not optimal).

Clarity

Central terms like "neural activity" are insufficiently defined. The study would gain from more stringent use of terminology - e.g., correlates, coding, encoding etc are loosely and interchangeably used, and are not defined (see further below). Clear definitions and transparency in data selection criteria are critical for reproducibility and interpretation.

Consistency

We find inconsistencies that are difficult to scientifically comprehend. This includes the rationale for administering optogenetic stimulation during the choice period (Fig. 6) given the general focus on the fixation period. We note a lack of accounting for or discussion regarding this.

There are unjustified shifts in the focus and analysis between figures, e.g., from perseverance to repeat probability.

Presentation

The ms holds statements not backed up by presentation of data and statistics. Examples of this:

Row 180: '...we found units in all brain regions whose activity correlated with choice and feedback after these events had begun'. No data for this appears to be presented.

Row 181: 'However, neurons whose activity during the fixation period predicted the upcoming choice were rarer and appeared far more common in MOs than in any other region (Figure 3C-E)'. In Fig 3D an example session is shown. In Fig 3E the (sub)regions are shown, without disclosure of which data is plotted or why only these three regions were picked. The statement thus appears to be an overstatement.

Overinterpretation

Calling the optogenetic stimulation of an undisclosed territory of PV+ neurons in MOs "inactivation of MOs" is misleading. Furthermore, the authors make statements about activity in MOs and the "dorsolateral PFC" as a whole but effectively are just sampling parts. See also comments under 'Anatomical precision'. Regarding the optogenetics experiments, no control experiments with light but no opsin were included as far as we can see and the only panels (S8) validating the targeting are

not really possible to evaluate - the only information given is red markings - no visualization of probe tracts, opsin expression or other relevant information.

"Coding"/"encoding" are suggestive metaphors that when misused reflect overstatements. Furthermore, although a choice can be decoded from selected neurons and neuronal activity can be correlated to e.g., perseverance, this does not mean that a whole brain region is "coding" for choice/perseverance.

Figure quality
Clearer and more explanatory figure legends would improve accessibility.
Fig. 1I&H: The legend descriptions for I and H are confused.
Figure 2D: The x-axis label reads ‘RT minus mean RT of the session’ while figure legend D says it’s mean response time.
Fig. 3CD: Alignment of data to events (e.g., go cue or movement onset) is unclear.
Fig. 3D: Unclear what the data is z-scored to (whole recording, pre-cue period?)
Fig. 5A: The y-axis appears flipped. Block indications are lacking, so it is unclear when the blocks are changing (as it is supposed to change every 125-175 trials?, yet the perseverative variable appears to change on a shorter scale).
Fig. 5B: The meaning of the dots is unclear. Why do left-preferring units fire more during fixation when the next choice is right? Needs a better explanation, also in the text.
Fig. 7B: The laser indications are not really visible.

Additional comments
In the section related to the response time, was only the overall response time analyzed or also the latency of the response initiation?
The block of text (lines 42-51) repeats twice in the introduction.
Line 98 - first mentioning of the PR model without spelling out of the abbreviation?
Method section (732-733) says that the Neuropixels probes were lowered at the speed of ‘300 micrometers per second’ - this appears unorthodox.
Line 852 says ‘in the same AP and DV location’, is AP and ML meant?
Panels that are supposed to be compared do not hold the same scale, e.g. S4B.

Version 1:

Reviewer comments:

Reviewer #1

(Remarks to the Author)
The authors have adressed my concerns up to my satisfaction.

Reviewer #2

(Remarks to the Author)
Thank you for your thorough and thoughtful response to the comments. Overall, the additional results integrate well with your original suggestions regarding the role of the secondary motor cortex (MOs), and the revised manuscript is acceptable in its current form. However, for the mPFC inactivation results presented in Figure 7, I believe that providing further interpretation would enhance the overall insightfulness of the paper. I suggest discussing these results in the context of previous studies on the role of the mPFC in reward learning. Additionally, if you do not plan to mention Supplementary Figure 22, it may be best to remove it; alternatively, you could refer to it in the discussion if it is relevant to your findings.

Reviewer #3

(Remarks to the Author)
I have no further comments - great job!

Reviewer #4

(Remarks to the Author)
We thank the authors for the actions performed in response to our comments, we this this had a positive effect on the integrity of the study. Nevertheless, we unfortunately find that the ms still holds clarity issues hindering assessment of the scientific validity of data and interpretations, conclusions, and claims. We here provide a very condensed account of our final impression of the study, with a few representative examples of key problem areas:

While the ms is improved, for example by defining what the authors understand as “activity”, it is still hard to comprehend what was actually done and what the data shows, partly due to insufficient precision and definitions of important concepts, and lack of consistency in terminology. As an example, ‘choice’ is used loosely, at times referring to a wheel turn in a specific direction and other times referring to something much more abstract. At times, choice appears to span/reflect a time period. This unspecificity negatively affects any analysis of correlations between choice and neuronal activity.
We find the ms to be continually troubled by many overinterpretations, including the title. We do not think that the study shows that the dorsal PFC drives perseverance. Instead, we think the study shows that there are neurons in the anterior

MOs whose activity correlates with the upcoming wheel turn direction and a preservative variable that can be inferred from behavior.
In line with this, we are not convinced that specifically the anterior MOs is responsible for perseveration, partly due to how units were spatially sampled and (anatomically) pooled - both can conceal the reality. The optogenetic experiments do not allow solid conclusions, particularly in light of the observed paradoxical rebound effect, that should be taken seriously and be incorporated into interpretation of the silencing experiments - there is no reason to think this effect should be specific to the ITI. Such rebound activity can cause unpredictable and obscure effects on MOs neural activity that go well beyond the “silencing” effect the authors claim. The optogenetic stimulation during the choice period might have a strong disturbing effect that directly influences motor behavior towards a wheel turn into a random direction. This would trivially explain why previous choices are not repeated.
Another problem is that signal-to-noise ratio can be assumed to correlate with firing rate. Hence, we expect better decoding performance from units with high firing rates. It appears necessary to check whether the improved decoding from MOs can trivially be explained by the MOs units displaying higher firing rates than other PFC regions. Figure 3c and d appear to suggest the MOs units do indeed display higher firing rates.
Lastly, the ms is still riddled with unfortunate sloppiness that has serious negative effects on the comprehension of the study, but would have been straightforward to avoid. Examples:
Methodological unclearities:
Inter-trial-intervals as well as inter-trial periods are mentioned and of importance. Lines 48-49 indicate that movement is not allowed during this epoch, while lines 508-09 indicate that movement is prohibited after the intertrial interval/period. One of the statements should be erroneous, and the reader is left to guess which.
Confusing and inconsistent terminology:
“Outcome period suppression” appears to at times be called ”Feedback time stimulation” - the mixing of outcome and feedback, and suppression and stimulation, respectively, is nothing but a source of confusion.
Fact errors:
Abstract states the brain region of focus to be supplementary motor cortex (MOs); it should arguably be secondary motor cortex (MOs).

Open Access This Peer Review File is licensed under a Creative Commons Attribution 4.0 International License, which permits use, sharing, adaptation, distribution and reproduction in any medium or format, as long as you give appropriate credit to the original author(s) and the source, provide a link to the Creative Commons license, and indicate if changes were made.
In cases where reviewers are anonymous, credit should be given to 'Anonymous Referee' and the source.
The images or other third party material in this Peer Review File are included in the article’s Creative Commons license, unless indicated otherwise in a credit line to the material. If material is not included in the article’s Creative Commons license and your intended use is not permitted by statutory regulation or exceeds the permitted use, you will need to obtain permission directly from the copyright holder.
To view a copy of this license, visit https://creativecommons.org/licenses/by/4.0/

Reviewer #1 (Remarks to the Author):

In this study, the authors examined the neural circuits of perseverative behaviour in Mice.

Using a probabilistic reversal task in head-fixed animals, the authors first examined the computational rules for performing in this task. They therefore compared the performance of 13 models using cross-validated likelihood and found that 5 models captured similar aspects of the mice behaviour. The authors chose the simplest one (so called perseverative/reward model, Miller, 2011) in order to fit the model with neural data.

Using Neuropixels probes, the authors then record neuronal activity in a set of forebrain structures, including the secondary motor area (MOs, available data for 15 sessions), the mPFC (available data for 14 sessions), the OFC (available data for 11 sessions), olfactory regions (available data for 15 sessions) and dorsal hippocampus (available data for 15 sessions). Using a set of sophisticated analysis, they found that the neural activity in MOs was the most predictive of the upcoming choice. Critically, they found that such an activity predicts the perseverative element of computation, but not the reward seeking element one. Finally, using optoinhibition, they found that inhibiting the MOs at the time of choice decreased perseverative behaviour.

Taken as a whole, this is a very well-written paper, which makes a very interesting contribution to the field. I have a few comments which are listed below.

1. The first comment is about the training

The authors state that the animals became expert in the task following 4 to 12 weeks of training. Could the authors provide some details for each mice about the acquisition of the task. It could also be very useful to be provided with a description of a possible relationship between the speed of learning and the amount of perserverative behaviour. Therefore, given that one might expect the animals strategy to gradually change with training, the authors should therefore provide evidence of such changes by providing training data.

Thank you for the suggestion, we have made a new figure (Supplementary Figure 4) that correlates the number of days of training required for each mouse to reach criterion vs. the behavior model parameters for each mouse. This shows no apparent correlation between the two variables. We note however that the judgement of an animal's proficiency in the task was made by an experimenter subjectively and was based on the number of trials and rewards collected during a session. We have included this caveat in the supplementary figure legend. A further study could

shed light on the relationship between the speed of learning and the perseverative behaviour. All training data will be released upon publication.

2. The second comment is about the targeted cortical areas and anatomical boundaries.

Previous research has demonstrated that choice in such task is regulated by different regions of the prefrontal cortex. Therefore, the authors should make clearer their rationale of exploring the role of the various brain regions recorded, in particular the DH and the olfactory regions. Also, the anatomical boundaries of the recorded areas should be better described given that important anatomical and functional dissociations have been described at the level of both the MOs and the mPFC. Importantly, current research has provided convincing evidence for a role of the OFC in adapting to change in contingencies in such a probabilistic task. Therefore, the lack of neural modulation in this brain region should be better discussed in relation to the well known role of the OFC in the Exploration/Exploitation trade-off and also win-stay/lose-shift behaviours.

Thank you, we have expanded the rationale for our choice of regions and clarified the locations within each that most of our units are from (lines 162-181 and elsewhere) . Our main targets were dorsal hippocampus (Masuda et al., 2020), striatum (Kim et al., 2009) and prefrontal cortex (Sul et al., 2009, Bari et al., 2019, Lee et al., 2012);we recorded in the olfactory areas serendipitously, because probes targeting the prefrontal cortex extended into them. Supplementary figures 8 and 10 show the distribution of the recorded units across the brain, and their correlates with choice during the movement and fixation periods.

With respect to orbital cortex, the rodent studies we are aware of studying a role in adapting to change in contingencies, managing explore/exploit, and win-stay/lose-shift all focus on LO and medial portion of AIv, which together are putatively homologous to primate OFC and often referred to as “rodent OFC” or sometimes “lateral OFC”. We did not record this area in the current study for methodological reasons related to the acute neuropixels prep: their lateral location would have required a outwardly-sweeping medial-to-lateral probe angle which would have made it difficult to simultaneously place additional probes in the brain, sharply limiting the throughput of our experiments. Instead, we report recordings from medial parts of the orbital cortex (MO and VO), which can readily be recorded simultaneously with mPFC using a single probe (Fig 3a). These regions are putatively homologous to primate vmPFC, and although they are sometimes referred to as “medial OFC”, we believe this term is misleading as they are not likely homologs of primate OFC. We have edited our text and figures to be more clear about this, referring to these regions now as “ORB” (E.g. lines 172-173).

These medial orbital regions we recorded from have not been studied nearly so extensively as the lateral ones homologous to primate OFC, and the studies we are aware of indicate that they may play a role in learning and decision-making only under very specific circumstances (Bradfield, et al., 2015; Bradfield, Hart, and Balleine, 2018; Gardner et al., 2018; Münster et al., 2018). The only

studies we are aware of that investigate the role of such areas specifically in bandit tasks are in primates (Noonan et al, 2010, Noonan et al., 2017), where excitotoxic lesions found to cause no effect on either learning or decision-making (Rudebeck et al., 2017). We are therefore cautious to draw parallels between our results in ORB and other literature.

Reviewer #2 (Remarks to the Author):

This study suggests a role for the secondary motor cortex (MOs) in a dynamic reward-learning task as a driver of perseverative choice behavior. Prior to movement, MOs represented the upcoming choice and a perseverative variable which was estimated from a behavioral model. Optogenetic inactivation during the decision period, achieved by stimulating interneurons in MOs, reduced repeated choice behavior, thereby impairing value-based decision-making. A similar experiment in the prefrontal cortex (PFC), conducted during the outcome period and in subsequent trials, slowed learning of switching reward probabilities, with a significantly larger effect size compared to MOs. These results highlight distinct roles for MOs and PFC at different stages of value-based decision-making.

This study examined choice behavior by analyzing two key variables: choice-related (perseverative, P) and value-related (reward-seeking, R) factors, proposing another view for interpreting behavior in a dynamic reward-learning task. Plus, although previous rodent studies (Kim et al., 2009; Sul et al., 2010; Sul et al., 2011; Lee et al., 2012; Bari et al., 2019; Hattori et al., 2019) demonstrated that most of the regions (excluding olfactory areas) recorded in this study signaled choice and/or value variables, such as action- and chosen-value, this study examined the neural representation of perseverative and reward-seeking variables. Additionally, while earlier studies showed that lesions in MOs in rats (Sul et al., 2011) and pharmacological inactivation of PFC in mice (Bari et al., 2019) impaired value-based choice behavior, this study investigated the specific stages within each trial and across trials that depend on the involvement of these regions.

However, to fully integrate and build upon the findings of this study, several points require further clarification.

1) Decoding of choice, perseverative variable (P) and reward-seeking variable (R), relate to Figure 4A

The fixation period may warrant further focus as it excludes motor responses and allows investigation into the aspect of planning. In the optogenetic experiment, since the laser was applied after the go cue (and not during the fixation period), it is important to examine neural representations during that period. While decoding results were presented after movement onset,

have you examined the decoding results for the period between the fixation offset and movement onset? Given the potential variability in the length of this period, focusing on data from around the fixation period, movement onset, or presenting analyses similar to those in Supplementary Figures 6 and 7 could provide additional insights.

Thank you, we have repeated these analyses on the periods following the go cue and the periods preceding the movement (Supplementary Figure 12). In more than half the trials movement occurs by 1s after the go cue, therefore we focused our analysis on that epoch. Our analysis showed that during the first 200 ms after the go cue, neural activity in only MOs significantly encoded the movement, whereas all other areas encoded it starting at later time bins. In all areas it was possible to decode the movement from the epoch 200-0 ms prior to movement execution, but decoding prior to this epoch was only reliable from MOs activity.

These new analyses complement our original results and further support our conclusions. The mPFC does not code the choice variable prior to the go cue (Figure 4), and only begins to encode the choice variable 200 ms after the go cue (Supplementary Figure 12), and inactivating mPFC after the go cue does not change the decision (Figure 6). In contrast, the choice variable is encoded in MOs activity prior and after the go cue (Figure 4, Supplementary Figure 12), and MOs inactivation after the go does change the decision (Figure 6). This indicates that MOs continues to carry a signal related to future movement throughout the period in which we optogenetically silenced, while for the bulk of this period mPFC does not. This supports the conclusion that MOs, but not mPFC, plays a causal role in the decision.

2) Neural representation of decision variable (P+R), related to Figure 5C

Similarly to the above point, have you examined the temporal pattern of neural representation for P+R (which may serve as the decision variable in this study) as shown in Supplementary Figures 6 and 7? The optogenetic experiment in MOs during the decision period decreased the weight of previous reward-choice interactions (Figure 6I), yet the reward-seeking variable does not appear to be represented in MOs (Figure 4A). Additionally, since the laser was not applied during the fixation period, it would be important to analyze neural representation after the matching period.

Thank you, we repeated the decoding analysis for the P+R variable (Supplementary Figure 14). MOs was the only area where it was possible to decode this variable throughout the 1s period following the go cue. It was possible to decode it in the orbital area in some time bins (200ms following the go cue, 800ms following the go cue, 1s following the go cue). In dorsomedial striatum and orbital area it was possible to decode the P+R variable in the timebin immediately prior to movement initiation. This indicates that neural activity in MOs, but not mPFC, correlated with P+R during the time of our optogenetic silencing. This is broadly similar to the pattern of results seen by analyzing P on its own, consistent with the fact that P and P+R are highly correlated.

3) Result of optogenetic experiment in PFC, related to Figure 6

Similar to MOs, how should we interpret the results of the optogenetic experiments in the PFC in relation to its neural representation during this task? During the outcome period, the reward-seeking variable is likely important for updating variables relevant to the next choice. However, the level of its representation appears to be stronger in MOs compared to PFC (Supplementary figure 7). Alternatively, if it is possible to examine updating-related variables in the model used in this study, such as reward prediction error or updated chosen-value from a Q-learning model that dynamically changes during transitions, could these variables be more prominently represented in PFC than in MOs?

In this context, considering or comparing the results of Bari et al. (2019), which demonstrated stable representations of decision variables from the Q-learning model in the PFC that are critical for value-based choice, with findings of this study could provide additional insights. Such comparisons might further highlight the distinct contributions of this study and provide more comprehensive information to readers.

Thank you, we have added an analysis of RPE decoding, defining the RPE as the actual reward minus the Q variable of a Q-learning model (Supplementary figure 22). The correlates of RPE are similar to those of reward itself (Supplementary Figures 17 and 18): found strongly in all recorded regions following reward outcome.

Our data do appear different to the results of Bari et al. We hypothesize this difference is due to differences in the task, as with the difference to Sul et al. We now discuss this further in the Discussion section (Line 432-440).

4) Comparison with previous studies, related to Discussion (line 379 – 389)

In Sul et al. (2011), lesions of the MOs increased lose-stay behavior but decreased the inverse temperature parameter in the Q-learning model, suggesting that switch or stay behavior may depend on previous choices and rewards. In this study, have you examined lose-switch behavior and model parameters, particularly those related to perseveration?

Additionally, experimental details such as precise reward schedules and head fixation vary across this study and earlier works (Sul et al., 2011; Hattori et al., 2019). In particular, the ratio of reward probabilities between the two rewarding sides is larger in this study compared to previous ones. These methodological differences may contribute to variations in observed choice behavior.

We have computed the lose-switch probability on trials with laser vs no laser (Supplementary figure 19). We found that the lose switch behaviour increased upon the laser inactivation of MOs at the go cue time. Thus, consistent with our other analyses, our results are precisely the opposite of Sul et al.

As to why, we agree with the reviewer: there are many methodological details that differ between our task and that of Sul et al, which probably explains the difference in results. We are not certain which of these differences is responsible, but speculate that the primary reason is the difference between spatial t-maze task (in which rats naturally alternate) and head-fixed choice tasks (in which mice naturally perseverate). We have elaborated on this in the discussion (lines 419-431).

5) Minor descriptive suggestions

Regarding Figure 1F (lines 88–89), have you tested for statistical differences between the weights of choice and those of reward-choice interactions? Additionally, interpretation or discussion related to Supplementary Figure 4B appears to be missing. Lastly, Supplementary Figure 9 should be referenced in the corresponding descriptions for clarity and completeness.

Thank you, we have added this statistical comparison for Figure 1F. We find that indeed the weights of choice are significantly different from the weights of the reward-choice interactions.

We have added a discussion of Supplementary Figure 4B (now Supplementary Figure 5B; lines 278-279) and Supplementary Figure 9 (now Supplementary Figure 20; lines 324-326), thank you for catching that omission.

Reviewer #3 (Remarks to the Author):

Lebedeva et al measured activity in multiple cortical brain regions during a task shown to produce perseverative behavior in mice, then optically inhibited selected brain regions to determine if this activity was causally linked to perseverative behavior. They observed that dorsal PFC activity predicts both perseverative behavior and response times, and inactivation of this region reduced perseveration. The technical aspects of this paper are excellent, and I admired the rigorous testing of different computational behavior models as well as the concise summaries of these approaches in the methods. I have some relatively minor concerns about interpretation, use of male/female mice, and the unclear criterion used for task comprehension, which are detailed below.

1. Further discussion of the cause of MO driving of “perseverative behavior” is warranted, as perseverative choice could reflect different scenarios. For example, perseveration may be a result of non-outcome driven, stimulus-response/habitual behavior. Alternatively, perseveration could be use of an exploitation over exploration strategy, with MO inactivation shifting mice toward increased exploration. Please discuss briefly in intro or discussion.

Thanks for pointing this out. We have revised the manuscript to more clearly differentiate “perseverative behavior” (the empirical observation that choices are autocorrelated to a degree not well-explained by reward-learning models) from “perseveration” (the hypothesis that this is due to a Law of Effect or habitual learning process by which past choices are causal to future choice). You are right to point out the alternative hypothesis that mice solve the task using a strategy that involves discrete “explore” and “exploit” states (Ebitz et al., 2018). We have expanded on this in the discussion. (Lines 406-418)

2. Unless I missed it, there is no mention of sex in this report. Please add this information, and provide rationale if only one sex was chosen.

Thank you, we have added this information in the methods section (Line 471).

3. The authors report that MO units encode direction of upcoming choice – can you provide evidence that this is not solely a spatial left vs. right signal, and instead has some link to goal-directed actions/outcome expectancy?

We were not sufficiently clear: we actually do think these are spatial signals, rather than related to goal-directed action or to outcome expectancy. Specifically, we find that fixation-period signals in MOs which correlate with upcoming choice are best-explained not as related to the binary upcoming choice per se, but to the perseverative decision variable which drives it (Fig 5). This perseverative decision variable is by definition not related to outcome expectancy but to choice history. We find no evidence that MOs signals are related to the reward-seeking decision variable (Fig 5C), which is the sole carrier of outcome-related information. Our findings are therefore more consistent with a role for these signals in “habitual” or “value-free” decision-making, rather than “goal-directed” or “value-based”. We have updated the descriptions of this in our Results and Discussion sections to be more clear about this (Lines 244-246, 415-418, 269-273).

4. In “Behavior Training”, it is unclear what criterion was used to determine that mice were trained in the task. Please provide more details – this is very important, as choice behavior in some subjects might reflect inability to discriminate between options rather than perseverative choice.

This judgement was made subjectively however all mice had at least 26 behavioural training sessions (34.4 ± 4.09, mean ± S.E.M., n=10 mice) by the time recordings started, and we have

added a figure to show that the amount of time trained did not correlate with perseverative behavior (Supplementary Figure 4). We now state this in the Methods (Lines 520-524)

5. In the “optimistic Q learning” section of methods, the term “negative reinforcement” is used incorrectly – in the instrumental literature, this term refers to performing an action to remove an aversive stimulus (rather than an action no longer being reinforced as in this experiment).

Thank you for pointing this out. We have corrected this section, which now refers to “positive and negative prediction errors” (Line 552).

6. When using optogenetics, it is good practice to include an optical stimulation condition during the ITI to control for non-specific effects (i.e. effects of optical inhibition might not be related to manipulation of event-specific activity, but instead caused by longer-lasting effects of inhibition on overall activity). Please add a brief explanation for why this is not a concern (I think the diverging effects of MO inhibition during different behavioral events should be sufficient to explain this).

Thank you. We believe that inactivation in the outcome period serves as an internal control for this, and have added a discussion of this point (Lines 328-331)

Reviewer #4 (Remarks to the Author):

I reviewed the ms by Lebedeva et al. together with two lab members working on very related topics and methodology.

The authors investigate the behavior and brain activity of mice engaged in a probabilistic two-choice reversal learning task using a combination of behavioral modeling, electrophysiology, and optogenetics. The study states to address which brain regions carry neural activity that correlates with, and has a causal role in, perseverative learning and decision-making. Whether perseveration is supported by a similar set of brain regions and internal cognitive variables as reward-guided learning and decision-making is also addressed.

A model was fit to the behavioral data, deriving behavioral variables representing perseverance (repeating previous choices) and reward-based learning.

Findings:

behavioral analysis: Mice persevere more than would be optimal for reward-maximization.

neuronal activity: Measuring neuronal activity in several brain regions extracellularly using Neuropixels, the authors report that choice and perseverance can be best decoded from (correlates best with) activity in the MOs.

optogenetic manipulation: Driving PV+ neurons in the MOs during the choice period reduced task performance and perseverance. Stimulating PV+ neurons in the “mPFC” after reward delivery impaired reward-based learning and caused slower adaptation to rule changes.

The authors conclude that MOs "encodes" the perseverative behavioral variable (or "drives preservation") rather than reward-optimization.

REVIEW SUMMARY

Strengths: Promising approach and data

In combining behavioral modeling, electrophysiology, and optogenetics the study employs a promising approach. The focus on perseverance, a relatively underexplored behavioral variable, adds novelty and the dataset appears robust, enabling a potentially nuanced exploration of the relationship between neuronal activity and decision behavior. The thorough behavioral analyses are a strength and provide important and interesting insights into the law of exercise and perseverative behavior.

Weakness: scientific rationale and data presentation

The study is quite straightforward - one behavior and a few, complementary and relevant methods. However, the presentation of the study does not reflect this and the rationale behind what is analyzed and presented versus not is enigmatic. The presentation gives the sense of an agenda different from a wish to truly investigate neuronal underpinnings of perseverative learning and decision-making, reducing the potential impact of the study.

The recorded neuronal activity is arguably the most important part of the study and a good amount of work should have gone into collecting this data (>19,000 units in the final dataset, however, the rationale behind the choice of brain subregions is unclear). It is therefore a mystery why not more effort and focus are put into analysis and presentation of this dataset. Concerning the actual experiments, we find the lack of spatial precision in analyses and presentation troublesome, hindering true insight into the circuits of perseverance. Beyond the selection of what is presented, we unfortunately find shortcomings in the way data and methods are presented and discussed (see further below).

Our overall view: the study addresses an interesting topic with a potentially suitable approach. However, substantial issues in manuscript organization, data selection and presentation, and explanation of methodology hinder a judgment of the study's scientific quality and reproducibility. These issues should be addressed before comments on specific revisions can be made.

Major points outlining what we find problematic

Manuscript organization

We find that the data representation in the manuscript is unnecessarily complicated and puzzling. Figure legends, while being wordy, offer little aid for comprehension. The ms manages to be shallow and complicated at the same time, which we think stems from the disconnect between what is said to be the focus of the study to what is actually analyzed/presented. There is a disproportion between what is given space versus not, with the lack of presentation of central data compromising the value of the study. While we find the behavioral analysis powerful, we find the neuronal activity to be neglected. Much of the analysis focuses on small subsets of neurons, with panels displaying example neurons, obscuring a view of the activity involved in perseverative learning and decision-making. This also gives a troubling flair of cherry-picking and contributes to the questioning of the study's scientific foundation and goal.

The Results section includes information that is better suited to the Methods section (e.g., detailed explanations of nonsense correlations, comparisons of similar behavioral models, Fig. 1G). Conversely, aspects that are arguably central to understanding and evaluation of the scientific content are buried in the Methods section, e.g. what the authors mean by "neural activity".

Thank you, we have reorganized the manuscript throughout following these suggestions.

Data validity

Anatomical precision

First, the scientific rationale behind what anatomical territory is recorded is unclear - the selection appears rather random. Furthermore, mapping of the anatomical position of Neuropixels recording sites is standard, e.g., using SHARP-Track, giving insight into 3D anatomical locations, layers etc.

In the current study, anatomy is overall neglected, both in analysis and the interpretation of the data. The MOs encompasses a very vast anatomical territory, and the authors make claims about MOs as a whole while in reality likely sampling and analyzing a small sub-territory - we find this to be a serious problem. Overall, it is unclear where specifically the activity originates, as the provided information and figures are very superficial. Several PFC subregions are lumped into mPFC, without a clear scientific motivation. Lumping of subregions (into PFC/HPC/ORB/olfactory etc.) cannot be justified given the current state of the art and does not reflect the frontline of the field. Beyond the unnecessary inaccuracy lumping entails, it also calls some of the analyses into question. We e.g. wonder how it affects the selection of random neurons. On a more basic level, we wonder how the neurons in the whole dataset correlate with the behavioral variable, where exactly are the highest correlations found, how correlations vary across anatomical 3D space etc.?

Thank you for raising this very important point. You are absolutely correct that the area of MOs we find to encode the variable is a specific subregion, and that we did not make full use of the spatial information about the recorded units. To address these we have specified that the region in question is a specific part of anterior MOs (Lines 9, 12-15, 48, 50, 56) that has previously been implicated in similar tasks (Lines 168-171). For brevity, we do at times use the unqualified term "MOs" to refer to this subregion, but have now repeatedly made it clear that this specifically should be taken to mean this anterior subregion (Lines 168-171, 221, 370)

We also now show maps of all recorded units, color coded to show where they had a significant correlation with choice during the fixation period (Supplementary figure 8) or movement period (Supplementary figure 9), allowing readers to form their own judgements about which regions do and do not correlate with task variables.

Nevertheless, we remain cautious about drawing strong conclusions about further precise anatomical regions, out of conservatism. First, even though we record a large number of neurons from each area, this number does not provide an "N" for statistical analysis - neurons recorded simultaneously are not statistically independent, and to draw rigorous statistical conclusions requires multiple recordings of each region. This is why the analysis methods in the main text are restricted to a smaller number of broad regions which we have recorded multiple times. Second, drawing distinctions between nearby areas (such as prelimbic and infralimbic cortex) is not always possible with high confidence, due to potential errors in probe localization.

We hope that the combination of showing the new map of all neurons, cautious statistical analysis, and further discussion (e.g. regarding the subregion of MOs) addresses this important point raised by the reviewer.

In terms of anatomy, we find that the authors do a disservice to both the study and the research question by treating brain regions as if they held entirely separate functions. This approach might

be particularly misleading for the PFC, as extensive data show shared connectivity between its regions, with gradual rather than abrupt shifts in connectivity (see eg Gao et al., 2022, DOI: 10.1038/s41593-022-01041-5). For example, some areas of the MOs may exhibit more similar connectivity to adjacent medial mPFC regions than to distant parts of the MOs - this would have implications on functon(s). A more unbiased, data-driven approach to analyzing the recorded activity and its correlation to behavioral variables would significantly strengthen the study.

Thank you, we absolutely agree. To address this point we have included maps of all correlated neurons to address this point (supplementary Figures 8 and 9). Many additional questions might be addressed by further analysis of this dataset, including questions related to detailed subregions within mPFC. To facilitate this we will make the data and code available online.

Neuronal activity: definition and selection process

The selection process for which units to include (only considering the 20 single units whose "activity" is most correlated to a behavioral variable) is not justified. Furthermore, it appears 20 units are selected based on their individually strong correlations with e.g. the behavioral variable. However, 20 units that are by themselves not so correlated with the behavioral variable could potentially TOGETHER predict the behavioral variable better. It is to be expected that the units that are individually very correlated to the behavioral variable are also very much correlated among themselves (so from an information perspective, the selection process is not optimal).

We assume you are referring to Figure 4, where we predict behavioral variables from neural activity. The method of selecting 20 neurons was a way to perform sparse regularization that we found to be effective, but we were not sufficiently clear about the motivation for this analysis, so have added a comparison of this strategy to a more commonly-used statistical regularization method (LASSO; Supplementary Figure 15). Unregularized multiple regression is not suitable in this case, because the large number of neurons recorded leads to overfitting. Furthermore, ridge regression based on L2 weight penalty is not optimal as it produces a large number of non-zero weights, but information in cortex is encoded by sparse sets of neurons. A common approach for sparse regression is LASSO, but we found that the strong penalties required to obtain good performance also led to underestimated weight magnitudes.

The approach we settled on is a simplification of stepwise regression, in which a sparse subset of predictive neurons is first identified, and weights fit to these neurons without penalty. We realize this approach is unconventional, and thus now include an analysis using LASSO regression (supplementary figure 15). LASSO produces a similar conclusion to our main results: coding is

stronger in MOs than other regions, and the P variable could be decoded both during the fixation period and throughout 1s after the movement initiation in MO. This new analysis also shows that the 20-best approach is more statistically powerful than LASSO, which was unable to significantly decode the P variable from the activity in mPFC or hippocampus after the movement has started. We therefore retained 20-best as the analysis method for our main-text figures.

It is of course possible that the decoding approach could be improved further, for example taking into account cell-cell correlations as the reviewer suggests. Nevertheless we believe the current method suffices for our current scientific questions. Regarding the choice of 20 units, this was made because it gave good predictions. We realize it might appear arbitrary, and had partially addressed this concern already - Figure 4B shows the analysis results are robust to the number of units considered, but only for choice decoding. We have now added additional analyses of decoding quality vs. # units for the other decoded variables, which again indicates robustness to this analysis parameter (Supplementary figure 11).

Clarity

Central terms like "neural activity" are insufficiently defined. The study would gain from more stringent use of terminology - e.g., correlates, coding, encoding etc are loosely and interchangeably used, and are not defined (see further below). Clear definitions and transparency in data selection criteria are critical for reproducibility and interpretation.

Thank you, we fully agree that unambiguous definitions are essential, and have endeavored to provide them throughout. Specifically, we define “activity” or “neural activity” to mean spike counts in a window of fixed length, and have added further definitions (Lines 183, 200-201). We previously used “correlates”, “coding” and “encoding” as synonyms, but now stick to “correlates”. The words “coding” and “encoding” no longer appear in the manuscript.

Consistency

We find inconsistencies that are difficult to scientifically comprehend. This includes the rationale for administering optogenetic stimulation during the choice period (Fig. 6) given the general focus on the fixation period. We note a lack of accounting for or discussion regarding this.

There are unjustified shifts in the focus and analysis between figures, e.g., from perseverance to repeat probability.

Thank you for raising this important point. We attempted to address inconsistencies throughout by changes in terminology and further discussion throughout. We have attempted to reduce or explain

changes in focus between figures textually, although this has become more challenging due to the many new analyses added in supplementary material thanks to valuable suggestions of all four reviewers.

Regarding the choice of time window for optogenetic inhibition, we did evaluate inhibition during the ITI in pilot experiments, but found a paradoxical “rebound” effect: even on trials where the Go cue never came, it led to contralateral movements when optogenetic inhibition ceased. We suspect this is due to “rebound activity”, i.e. an increase in MOs spiking following the release from inhibition, and may thus provide an important clue to the neural mechanisms of the task, which is sufficient to drive a movement even when no Go cue is present. However to make confident claims about this phenomenon will require substantially more work, for example simultaneous recording with optogenetic inhibition, and we therefore prefer to leave it for future work.

Presentation

The ms holds statements not backed up by presentation of data and statistics. Examples of this:

Row 180: ‘...we found units in all brain regions whose activity correlated with choice and feedback after these events had begun’. No data for this appears to be presented.

We had already provided example units whose activity correlates with choice (figure 3C), and statistical analysis confirming choice after feedback onset in 6 regions (Figure 4A, top row). We have now added example units whose activity correlates with feedback (Supplementary figure 7A) and summary statistics of feedback coding (Supplementary figure 7B). In addition we now provide maps showing the spatial location of all neurons significantly correlating with choice before and after movement initiation (Supplementary figure 8, 9), and also correlating with feedback (Supplementary figure 10).

Row 181: ‘However, neurons whose activity during the fixation period predicted the upcoming choice were rarer and appeared far more common in MOs than in any other region (Figure 3C-E)’. In Fig 3D an example session is shown. In Fig 3E the (sub)regions are shown, without disclosure of which data is plotted or why only these three regions were picked. The statement thus appears to be an overstatement.

Thank you, we agree that figure 3 only provides examples, and that confirmatory statistics for this claim do not appear until figure 4. We have adjusted the text accordingly (Lines 182-188).

Overinterpretation

Calling the optogenetic stimulation of an undisclosed territory of PV+ neurons in MOs "inactivation of MOs" is misleading. Furthermore, the authors make statements about activity in MOs and the "dorsolateral PFC" as a whole but effectively are just sampling parts. See also comments under ‘Anatomical precision’. Regarding the optogenetics experiments, no control experiments with light but no opsin were included as far as we can see and the only panels (S8)

validating the targeting are not really possible to evaluate - the only information given is red markings - no visualization of probe tracts, opsin expression or other relevant information.

Thank you, as discussed in the response to comments in the “Anatomical precision” section of this review , we had previously used MOs to refer specifically to an anterior aspect of it, and have now corrected this throughout. We have also made it clear that we have inactivated only a portion of MOs and dlPFC (Lines 291-297). We also added an example histological image (supplementary figure 21A) showing cannula locations.

Regarding control experiments, we would argue that inactivation of MOs during the outcome period serves as an internal control, showing that the timing of inactivation mattered and that simply inactivating the region outside the decision window did not affect the choice. Inactivation of mPFC provides a second internal control, showing that the light itself or laser shutter sound was not sufficient to drive change in animal behaviour. We have included this discussion of control experiments in the text (lines 328-331)

"Coding"/"encoding" are suggestive metaphors that when misused reflect overstatements. Furthermore, although a choice can be decoded from selected neurons and neuronal activity can be correlated to e.g., perseverance, this does not mean that a whole brain region is "coding" for choice/perseverance.

This is a good point, related to your earlier point about careful definitions. In the previous version of the manuscript, we used the term “coding” simply to mean correlation - which is a common use of the term although not universal (e.g. deCharms and Zador Ann. Rev. Neurosci. 2000). We have now removed the terms “coding” and “encoding” altogether and stick to the word “correlation”.

Figure quality

Clearer and more explanatory figure legends would improve accessibility.

Thank you, we have rewritten the legends throughout.

Fig. 1I&H: The legend descriptions for I and H are confused.

Thank you, we addressed this.

Figure 2D: The x-axis label reads ‘RT minus mean RT of the session’ while figure legend D says it’s mean response time.

Thank you, we addressed this.

Fig. 3CD: Alignment of data to events (e.g., go cue or movement onset) is unclear.

We have clarified that the x-axis represents time in seconds relative to movement onset (in 3C) and relative to the Go cue in 3D.

Fig. 3D: Unclear what the data is z-scored to (whole recording, pre-cue period?)

Thank you, we adjusted the legend to be more precise.

Fig. 5A: The y-axis appears flipped. Block indications are lacking, so it is unclear when the blocks are changing (as it is supposed to change every 125-175 trials?, yet the perseverative variable appears to change on a shorter scale).

Thank you, we addressed this.

Fig. 5B: The meaning of the dots is unclear. Why do left-preferring units fire more during fixation when the next choice is right? Needs a better explanation, also in the text.

Thank you, this was a typo which we now fixed.

Fig. 7B: The laser indications are not really visible.

Thank you, we fixed this.

Additional comments

In the section related to the response time, was only the overall response time analyzed or also the latency of the response initiation?

Sorry that this wasn't clear. As mentioned in section "Perseverative choices are associated with faster responses" we quantified each trial's response time as the time between go cue onset and the moment when the wheel turn magnitude passed the threshold required to register a choice. Latency to response initiation is hard to define with wheel turn data. For example, on some trials mice started to turn the wheel to one side and then switched direction, and on some trials the initial wheel turn didn't lead to a full movement that registered as a choice. To avoid these complications we stuck to the simple definition, which is sufficient to answer our scientific questions. We now discuss this issue in the text and methods section (Lines 149-151, 511-512).

The block of text (lines 42-51) repeats twice in the introduction.

Thank you, we addressed this.

Line 98 - first mentioning of the PR model without spelling out of the abbreviation?

Thank you, we addressed this (Line 92).

Method section (732-733) says that the Neuropixels probes were lowered at the speed of ‘300 micrometers per second’ - this appears unorthodox.

Thank you, this was a typo which we now fixed (Line 789).

Line 852 says ‘in the same AP and DV location’, is AP and ML meant?

Thank you, this was a typo which we now fixed (Line 294, 910).

Panels that are supposed to be compared do not hold the same scale, e.g. S4B.

Thank you, we addressed this. Please note that due to change of figure numbers former figure supplementary figure 4B is now supplementary figure 5B

Reviewer #1 (Remarks to the Author):

The authors have adressed my concerns up to my satisfaction.

Thank you for your helpful review of our manuscript

Reviewer #2

Thank you for your thorough and thoughtful response to the comments. Overall, the additional results integrate well with your original suggestions regarding the role of the secondary motor cortex (MOs), and the revised manuscript is acceptable in its current form. However, for the mPFC inactivation results presented in Figure 7, I believe that providing further interpretation would enhance the overall insightfulness of the paper. I suggest discussing these results in the context of previous studies on the role of the mPFC in reward learning.

Thank you for your helpful review and positive assessment of our work. We agree that further discussing our mPFC inactivation results would add to the insightfulness of our paper. We have added a brief paragraph discussing similarities and differences with other similar results in the literature (Lines 434-444).

Additionally, if you do not plan to mention Supplementary Figure 22, it may be best to remove it; alternatively, you could refer to it in the discussion if it is relevant to your findings.

Thank you, we have removed the figure showing the RPE coding.

Reviewer #3 (Remarks to the Author):

I have no further comments - great job!

Thank you for your helpful review of our manuscript

Reviewer #4

We thank the authors for the actions performed in response to our comments, we this this had a positive effect on the integrity of the study. Nevertheless, we unfortunately find that the ms still holds clarity issues hindering assessment of the scientific validity of data and interpretations, conclusions, and claims. We here provide a very condensed account of our final impression of the study, with a few representative examples of key problem areas:

While the ms is improved, for example by defining what the authors understand as “activity”, it is still hard to comprehend what was actually done and what the data shows, partly due to insufficient precision and definitions of important concepts, and lack of consistency in terminology. As an example, ‘choice’ is used loosely, at times referring to a wheel turn in a specific direction and other times referring to something much more abstract. At times, choice appears to span/reflect a time period. This unspecificity negatively affects any analysis of correlations between choice and neuronal activity.

Thank you. We have corrected the manuscript to make sure that the word “choice” refers only to a technical definition: a binary variable given by the specific conditions required to register a choice in the current study, a wheel turn in a specific direction by 18mm in either direction (defined on lines 64, 518). We define the phrase "choice period” as the time from the go cue until the choice is registered, or 4.8s, whichever is faster (as defined on lines 298-299 of the manuscript).

Thus we have now given “choice” a strictly technical meaning as appropriate specifically for this behavior: when we need to refer to more abstract notions (e.g. when referring to other tasks in the abstract, introduction, and discussion), we now use the term “action” instead.

We hope this has removed any avenue of possible confusion for readers in this matter.

We find the ms to be continually troubled by many overinterpretations, including the title. We do not think that the study shows that the dorsal PFC drives perseverance. Instead, we think the study shows that there are neurons in the anterior MOs whose activity correlates with the upcoming wheel turn direction and a preservative variable that can be inferred from behavior.

In line with this, we are not convinced that specifically the anterior MOs is responsible for perseveration, partly due to how units were spatially sampled and (anatomically) pooled - both can conceal the reality. The optogenetic experiments do not allow solid conclusions, particularly in light of the observed paradoxical rebound effect, that should be taken seriously and be incorporated into interpretation of the silencing experiments - there is no reason to think this effect should be specific to the ITI. Such rebound activity can cause unpredictable and obscure effects on MOs neural activity that go well beyond the “silencing” effect the authors claim. The optogenetic stimulation during the choice period might have a strong disturbing effect that directly influences motor behavior towards a wheel turn into a random direction. This would trivially explain why previous choices are not repeated.

Regarding the rebound effect, for the great majority of opto trials (84%) the choice happens before the stimulation ends, so rebound effect is not an issue. This point was previously stated in the previous version of the text, but we hope that including it also in a new supplementary figure 22 will help make this clear to readers who might otherwise have missed it. We have also repeated the original analysis excluding all trials where choice happens after the stimulation is over and our conclusion remains the same (supplementary figure 22).

Regarding the more general point of optogenetic disturbance, mPFC inactivation provides an internal control for this. If optogenetic stimulation had a general but non-specific disturbing effect, then mPFC inactivation would have a similar effect to MOs inactivation. However, as figures 6 and 7 show, the effects depend on area. Only MOs inactivation affects perseveration, whereas mPFC affects feedback-based learning. This double dissociation of the effects of inactivating the two areas shows the effect cannot be due to general disturbance.

We have clarified the text to emphasize this point further (line 362-364).

Another problem is that signal-to-noise ratio can be assumed to correlate with firing rate. Hence, we expect better decoding performance from units with high firing rates. It appears necessary to check whether the improved decoding from MOs can trivially be explained by the MOs units displaying higher firing rates than other PFC regions. Figure 3c and d appear to suggest the MOs units do indeed display higher firing rates.

Figure 3c and d show example units. We have now added a supplementary figure 23 showing the firing rates for each region during the 200ms fixation period. This figure shows that there was no significant difference between the firing rates in MOs and all other areas.

Lastly, the ms is still riddled with unfortunate sloppiness that has serious negative effects on the comprehension of the study, but would have been straightforward to avoid. Examples:
Methodological unclearities:
Inter-trial-intervals as well as inter-trial periods are mentioned and of importance. Lines 48-49 indicate that movement is not allowed during this epoch, while lines 508-09 indicate that movement is prohibited after the intertrial interval/period. One of the statements should be erroneous, and the reader is left to guess which.

Thank you, lines 48-49 contained a typo which we now corrected.

Confusing and inconsistent terminology:
“Outcome period suppression” appears to at times be called ”Feedback time stimulation” - the mixing of outcome and feedback, and suppression and stimulation, respectively, is nothing but a source of confusion.

Respectfully, we don't see this as a source of confusion. Feedback and outcome are synonymous in this context and we clearly explain that our optogenetic stimulation is inhibitory.

Fact errors:
Abstract states the brain region of focus to be supplementary motor cortex (MOs); it should arguably be secondary motor cortex (MOs).

Thank you, we have corrected this.
